# Exploring age and gender disparities in cardiometabolic phenotypes and lipidomic signatures among Chinese adults: a nationwide cohort study

**DOI:** 10.1093/lifemeta/loae032

**Published:** 2024-08-02

**Authors:** Xiaojing Jia, Hong Lin, Ruizhi Zheng, Shuangyuan Wang, Yilan Ding, Chunyan Hu, Mian Li, Yu Xu, Min Xu, Guixia Wang, Lulu Chen, Tianshu Zeng, Ruying Hu, Zhen Ye, Lixin Shi, Qing Su, Yuhong Chen, Xuefeng Yu, Li Yan, Tiange Wang, Zhiyun Zhao, Guijun Qin, Qin Wan, Gang Chen, Meng Dai, Di Zhang, Bihan Qiu, Xiaoyan Zhu, Jie Zheng, Xulei Tang, Zhengnan Gao, Feixia Shen, Xuejiang Gu, Zuojie Luo, Yingfen Qin, Li Chen, Xinguo Hou, Yanan Huo, Qiang Li, Yinfei Zhang, Chao Liu, Youmin Wang, Shengli Wu, Tao Yang, Huacong Deng, Jiajun Zhao, Yiming Mu, Shenghan Lai, Donghui Li, Weiguo Hu, Guang Ning, Weiqing Wang, Yufang Bi, Jieli Lu, for the 4C Study Group, Guang Ning, Yiming Mu, Jiajun Zhao, Weiqing Wang, Chao Liu, Yufang Bi, Donghui Li, Shenghan Lai, Zachary T. Bloomgarden, Jieli Lu, Mian Li, Lulu Chen, Lixin Shi, Qiang Li, Tao Yang, Li Yan, Qin Wan, Shengli Wu, Guixia Wang, Zuojie Luo, Yingfen Qin, Xulei Tang, Gang Chen, Yanan Huo, Zhengnan Gao, Qing Su, Zhen Ye, Ruying Hu, Youmin Wang, Guijun Qin, Huacong Deng, Xuefeng Yu, Feixia Shen, Li Chen

**Affiliations:** Department of Endocrine and Metabolic Diseases, Shanghai Institute of Endocrine and Metabolic Diseases, Ruijin Hospital, Shanghai Jiao Tong University School of Medicine, Shanghai 200025, China; Shanghai National Clinical Research Center for Endocrine and Metabolic Diseases, Key Laboratory for Endocrine and Metabolic Diseases of the National Health Commission of the People's Republic of China, Shanghai National Center for Translational Medicine, Ruijin Hospital, Shanghai Jiao Tong University School of Medicine, Shanghai 200025, China; Department of Endocrine and Metabolic Diseases, Shanghai Institute of Endocrine and Metabolic Diseases, Ruijin Hospital, Shanghai Jiao Tong University School of Medicine, Shanghai 200025, China; Shanghai National Clinical Research Center for Endocrine and Metabolic Diseases, Key Laboratory for Endocrine and Metabolic Diseases of the National Health Commission of the People's Republic of China, Shanghai National Center for Translational Medicine, Ruijin Hospital, Shanghai Jiao Tong University School of Medicine, Shanghai 200025, China; Department of Endocrine and Metabolic Diseases, Shanghai Institute of Endocrine and Metabolic Diseases, Ruijin Hospital, Shanghai Jiao Tong University School of Medicine, Shanghai 200025, China; Shanghai National Clinical Research Center for Endocrine and Metabolic Diseases, Key Laboratory for Endocrine and Metabolic Diseases of the National Health Commission of the People's Republic of China, Shanghai National Center for Translational Medicine, Ruijin Hospital, Shanghai Jiao Tong University School of Medicine, Shanghai 200025, China; Department of Endocrine and Metabolic Diseases, Shanghai Institute of Endocrine and Metabolic Diseases, Ruijin Hospital, Shanghai Jiao Tong University School of Medicine, Shanghai 200025, China; Shanghai National Clinical Research Center for Endocrine and Metabolic Diseases, Key Laboratory for Endocrine and Metabolic Diseases of the National Health Commission of the People's Republic of China, Shanghai National Center for Translational Medicine, Ruijin Hospital, Shanghai Jiao Tong University School of Medicine, Shanghai 200025, China; Department of Endocrine and Metabolic Diseases, Shanghai Institute of Endocrine and Metabolic Diseases, Ruijin Hospital, Shanghai Jiao Tong University School of Medicine, Shanghai 200025, China; Shanghai National Clinical Research Center for Endocrine and Metabolic Diseases, Key Laboratory for Endocrine and Metabolic Diseases of the National Health Commission of the People's Republic of China, Shanghai National Center for Translational Medicine, Ruijin Hospital, Shanghai Jiao Tong University School of Medicine, Shanghai 200025, China; Department of Endocrine and Metabolic Diseases, Shanghai Institute of Endocrine and Metabolic Diseases, Ruijin Hospital, Shanghai Jiao Tong University School of Medicine, Shanghai 200025, China; Shanghai National Clinical Research Center for Endocrine and Metabolic Diseases, Key Laboratory for Endocrine and Metabolic Diseases of the National Health Commission of the People's Republic of China, Shanghai National Center for Translational Medicine, Ruijin Hospital, Shanghai Jiao Tong University School of Medicine, Shanghai 200025, China; Department of Endocrine and Metabolic Diseases, Shanghai Institute of Endocrine and Metabolic Diseases, Ruijin Hospital, Shanghai Jiao Tong University School of Medicine, Shanghai 200025, China; Shanghai National Clinical Research Center for Endocrine and Metabolic Diseases, Key Laboratory for Endocrine and Metabolic Diseases of the National Health Commission of the People's Republic of China, Shanghai National Center for Translational Medicine, Ruijin Hospital, Shanghai Jiao Tong University School of Medicine, Shanghai 200025, China; Department of Endocrine and Metabolic Diseases, Shanghai Institute of Endocrine and Metabolic Diseases, Ruijin Hospital, Shanghai Jiao Tong University School of Medicine, Shanghai 200025, China; Shanghai National Clinical Research Center for Endocrine and Metabolic Diseases, Key Laboratory for Endocrine and Metabolic Diseases of the National Health Commission of the People's Republic of China, Shanghai National Center for Translational Medicine, Ruijin Hospital, Shanghai Jiao Tong University School of Medicine, Shanghai 200025, China; Department of Endocrine and Metabolic Diseases, Shanghai Institute of Endocrine and Metabolic Diseases, Ruijin Hospital, Shanghai Jiao Tong University School of Medicine, Shanghai 200025, China; Shanghai National Clinical Research Center for Endocrine and Metabolic Diseases, Key Laboratory for Endocrine and Metabolic Diseases of the National Health Commission of the People's Republic of China, Shanghai National Center for Translational Medicine, Ruijin Hospital, Shanghai Jiao Tong University School of Medicine, Shanghai 200025, China; Department of Endocrine and Metabolic Diseases, The First Hospital of Jilin University, Changchun, Jilin 130021, China; Department of Endocrine and Metabolic Diseases, Union Hospital, Tongji Medical College, Huazhong University of Science and Technology, Wuhan, Hubei 430022, China; Department of Endocrine and Metabolic Diseases, Union Hospital, Tongji Medical College, Huazhong University of Science and Technology, Wuhan, Hubei 430022, China; Zhejiang Provincial Center for Disease Control and Prevention, Hangzhou, Zhejiang 310051, China; Zhejiang Provincial Center for Disease Control and Prevention, Hangzhou, Zhejiang 310051, China; Department of Endocrine and Metabolic Diseases, Affiliated Hospital of Guiyang Medical College, Guiyang, Guizhou 550004, China; Department of Endocrine and Metabolic Diseases, Xinhua Hospital Affiliated to Shanghai Jiao Tong University School of Medicine, Shanghai 200092, China; Department of Endocrine and Metabolic Diseases, Shanghai Institute of Endocrine and Metabolic Diseases, Ruijin Hospital, Shanghai Jiao Tong University School of Medicine, Shanghai 200025, China; Shanghai National Clinical Research Center for Endocrine and Metabolic Diseases, Key Laboratory for Endocrine and Metabolic Diseases of the National Health Commission of the People's Republic of China, Shanghai National Center for Translational Medicine, Ruijin Hospital, Shanghai Jiao Tong University School of Medicine, Shanghai 200025, China; Department of Endocrine and Metabolic Diseases, Tongji Hospital, Tongji Medical College, Huazhong University of Science and Technology, Wuhan, Hubei 430030, China; Department of Endocrine and Metabolic Diseases, Sun Yat-sen Memorial Hospital, Sun Yat-sen University, Guangzhou, Guangdong 510120, China; Department of Endocrine and Metabolic Diseases, Shanghai Institute of Endocrine and Metabolic Diseases, Ruijin Hospital, Shanghai Jiao Tong University School of Medicine, Shanghai 200025, China; Shanghai National Clinical Research Center for Endocrine and Metabolic Diseases, Key Laboratory for Endocrine and Metabolic Diseases of the National Health Commission of the People's Republic of China, Shanghai National Center for Translational Medicine, Ruijin Hospital, Shanghai Jiao Tong University School of Medicine, Shanghai 200025, China; Department of Endocrine and Metabolic Diseases, Shanghai Institute of Endocrine and Metabolic Diseases, Ruijin Hospital, Shanghai Jiao Tong University School of Medicine, Shanghai 200025, China; Shanghai National Clinical Research Center for Endocrine and Metabolic Diseases, Key Laboratory for Endocrine and Metabolic Diseases of the National Health Commission of the People's Republic of China, Shanghai National Center for Translational Medicine, Ruijin Hospital, Shanghai Jiao Tong University School of Medicine, Shanghai 200025, China; Department of Endocrine and Metabolic Diseases, The First Affiliated Hospital of Zhengzhou University, Zhengzhou, Henan 450052, China; Department of Endocrine and Metabolic Diseases, The Affiliated Hospital of Southwest Medical University, Luzhou, Sichuan 646000, China; Department of Endocrine and Metabolic Diseases, Fujian Provincial Hospital, Fujian Medical University, Fuzhou, Fujian 350003, China; Department of Endocrine and Metabolic Diseases, Shanghai Institute of Endocrine and Metabolic Diseases, Ruijin Hospital, Shanghai Jiao Tong University School of Medicine, Shanghai 200025, China; Shanghai National Clinical Research Center for Endocrine and Metabolic Diseases, Key Laboratory for Endocrine and Metabolic Diseases of the National Health Commission of the People's Republic of China, Shanghai National Center for Translational Medicine, Ruijin Hospital, Shanghai Jiao Tong University School of Medicine, Shanghai 200025, China; Department of Endocrine and Metabolic Diseases, Shanghai Institute of Endocrine and Metabolic Diseases, Ruijin Hospital, Shanghai Jiao Tong University School of Medicine, Shanghai 200025, China; Shanghai National Clinical Research Center for Endocrine and Metabolic Diseases, Key Laboratory for Endocrine and Metabolic Diseases of the National Health Commission of the People's Republic of China, Shanghai National Center for Translational Medicine, Ruijin Hospital, Shanghai Jiao Tong University School of Medicine, Shanghai 200025, China; Department of Endocrine and Metabolic Diseases, Shanghai Institute of Endocrine and Metabolic Diseases, Ruijin Hospital, Shanghai Jiao Tong University School of Medicine, Shanghai 200025, China; Shanghai National Clinical Research Center for Endocrine and Metabolic Diseases, Key Laboratory for Endocrine and Metabolic Diseases of the National Health Commission of the People's Republic of China, Shanghai National Center for Translational Medicine, Ruijin Hospital, Shanghai Jiao Tong University School of Medicine, Shanghai 200025, China; Department of Endocrine and Metabolic Diseases, Shanghai Institute of Endocrine and Metabolic Diseases, Ruijin Hospital, Shanghai Jiao Tong University School of Medicine, Shanghai 200025, China; Shanghai National Clinical Research Center for Endocrine and Metabolic Diseases, Key Laboratory for Endocrine and Metabolic Diseases of the National Health Commission of the People's Republic of China, Shanghai National Center for Translational Medicine, Ruijin Hospital, Shanghai Jiao Tong University School of Medicine, Shanghai 200025, China; Department of Endocrine and Metabolic Diseases, Shanghai Institute of Endocrine and Metabolic Diseases, Ruijin Hospital, Shanghai Jiao Tong University School of Medicine, Shanghai 200025, China; Shanghai National Clinical Research Center for Endocrine and Metabolic Diseases, Key Laboratory for Endocrine and Metabolic Diseases of the National Health Commission of the People's Republic of China, Shanghai National Center for Translational Medicine, Ruijin Hospital, Shanghai Jiao Tong University School of Medicine, Shanghai 200025, China; Department of Endocrine and Metabolic Diseases, The First Hospital of Lanzhou University, Lanzhou, Gansu 730000, China; Department of Endocrine and Metabolic Diseases, Dalian Municipal Central Hospital, Dalian, Liaoning 116033, China; Department of Endocrine and Metabolic Diseases, The First Affiliated Hospital of Wenzhou Medical University, Wenzhou, Zhejiang 325000, China; Department of Endocrine and Metabolic Diseases, The First Affiliated Hospital of Wenzhou Medical University, Wenzhou, Zhejiang 325000, China; Department of Endocrine and Metabolic Diseases, The First Affiliated Hospital of Guangxi Medical University, Nanning, Guangxi 530021, China; Department of Endocrine and Metabolic Diseases, The First Affiliated Hospital of Guangxi Medical University, Nanning, Guangxi 530021, China; Department of Endocrine and Metabolic Diseases, Qilu Hospital of Shandong University, Jinan, Shandong 250012, China; Department of Endocrine and Metabolic Diseases, Qilu Hospital of Shandong University, Jinan, Shandong 250012, China; Department of Endocrine and Metabolic Diseases, Jiangxi Provincial People’s Hospital Affiliated to Nanchang University, Nanchang, Jiangxi 330006, China; Department of Endocrine and Metabolic Diseases, The Second Affiliated Hospital of Harbin Medical University, Harbin, Heilongjiang 150086, China; Department of Endocrine and Metabolic Diseases, Central Hospital of Shanghai Jiading District, Shanghai 201800, China; Department of Endocrine and Metabolic Diseases, Jiangsu Province Hospital on Integration of Chinese and Western Medicine, Nanjing, Jiangsu 210028, China; Department of Endocrine and Metabolic Diseases, The First Affiliated Hospital of Anhui Medical University, Hefei, Anhui 230022, China; Department of Endocrine and Metabolic Diseases, Karamay Municipal People’s Hospital, Karamay, Xinjiang 834000, China; Department of Endocrine and Metabolic Diseases, The First Affiliated Hospital of Nanjing Medical University, Nanjing, Jiangsu 210029, China; Department of Endocrine and Metabolic Diseases, The First Affiliated Hospital of Chongqing Medical University, Chongqing 400016, China; Department of Endocrine and Metabolic Diseases, Shandong Provincial Hospital Affiliated to Shandong University, Jinan, Shandong 250021, China; Department of Endocrine and Metabolic Diseases, Chinese People’s Liberation Army General Hospital, Beijing 100853, China; Department of Epidemiology and Public Health, University of Maryland School of Medicine, Baltimore, MD 21201, United States; Department of Gastrointestinal Medical Oncology, The University of Texas MD Anderson Cancer Center, Houston, TX 77030, United States; Department of Geriatrics, Medical Center on Aging, Ruijin Hospital, Shanghai Jiao Tong University School of Medicine, Shanghai 200025, China; Department of Endocrine and Metabolic Diseases, Shanghai Institute of Endocrine and Metabolic Diseases, Ruijin Hospital, Shanghai Jiao Tong University School of Medicine, Shanghai 200025, China; Shanghai National Clinical Research Center for Endocrine and Metabolic Diseases, Key Laboratory for Endocrine and Metabolic Diseases of the National Health Commission of the People's Republic of China, Shanghai National Center for Translational Medicine, Ruijin Hospital, Shanghai Jiao Tong University School of Medicine, Shanghai 200025, China; Department of Endocrine and Metabolic Diseases, Shanghai Institute of Endocrine and Metabolic Diseases, Ruijin Hospital, Shanghai Jiao Tong University School of Medicine, Shanghai 200025, China; Shanghai National Clinical Research Center for Endocrine and Metabolic Diseases, Key Laboratory for Endocrine and Metabolic Diseases of the National Health Commission of the People's Republic of China, Shanghai National Center for Translational Medicine, Ruijin Hospital, Shanghai Jiao Tong University School of Medicine, Shanghai 200025, China; Department of Endocrine and Metabolic Diseases, Shanghai Institute of Endocrine and Metabolic Diseases, Ruijin Hospital, Shanghai Jiao Tong University School of Medicine, Shanghai 200025, China; Shanghai National Clinical Research Center for Endocrine and Metabolic Diseases, Key Laboratory for Endocrine and Metabolic Diseases of the National Health Commission of the People's Republic of China, Shanghai National Center for Translational Medicine, Ruijin Hospital, Shanghai Jiao Tong University School of Medicine, Shanghai 200025, China; Department of Endocrine and Metabolic Diseases, Shanghai Institute of Endocrine and Metabolic Diseases, Ruijin Hospital, Shanghai Jiao Tong University School of Medicine, Shanghai 200025, China; Shanghai National Clinical Research Center for Endocrine and Metabolic Diseases, Key Laboratory for Endocrine and Metabolic Diseases of the National Health Commission of the People's Republic of China, Shanghai National Center for Translational Medicine, Ruijin Hospital, Shanghai Jiao Tong University School of Medicine, Shanghai 200025, China

**Keywords:** sex difference, aging, metabolic diseases, modifiable risk factors, lipidomics

## Abstract

Understanding sex disparities in modifiable risk factors across the lifespan is essential for crafting individualized intervention strategies. We aim to investigate age-related sex disparity in cardiometabolic phenotypes in a large nationwide Chinese cohort. A total of 254,670 adults aged 40 years or older were selected from a population-based cohort in China. Substantial sex disparities in the prevalence of metabolic diseases were observed across different age strata, particularly for dyslipidemia and its components. Generalized additive models were employed to characterize phenotype features, elucidating how gender differences evolve with advancing age. Half of the 16 phenotypes consistently exhibited no sex differences, while four (high-density lipoprotein [HDL] cholesterol, apolipoprotein A1, diastolic blood pressure, and fasting insulin) displayed significant sex differences across all age groups. Triglycerides, apolipoprotein B, non-HDL cholesterol, and total cholesterol demonstrated significant age-dependent sex disparities. Notably, premenopausal females exhibited significant age-related differences in lipid levels around the age of 40–50 years, contrasting with the relatively stable associations observed in males and postmenopausal females. Menopause played an important but not sole role in age-related sex differences in blood lipids. Sleep duration also had an age- and sex-dependent impact on lipids. Lipidomic analysis and K-means clustering further revealed that 58.6% of the 263 measured lipids varied with sex and age, with sphingomyelins, cholesteryl esters, and triacylglycerols being the most profoundly influenced lipid species by the combined effects of age, sex, and their interaction. These findings underscore the importance of age consideration when addressing gender disparities in metabolic diseases and advocate for personalized, age-specific prevention and management.

## Introduction

With the aging of the global population, the prevalence of metabolic diseases has expandingly increased over the past two decades [1]. Global estimates revealed a substantial rise, with a total of 463 million cases of type 2 diabetes (T2DM) [2] and 1.28 billion cases of hypertension in 2019 [3]. Furthermore, in 2008, approximately 39% of adults aged 25 years and older exhibited elevated plasma total cholesterol (TC) levels [4]. Meanwhile, China grapples with the largest population aged 65 years and older [5] and a faster pace of population aging than many other countries [6]. This demographic shift contributes to the escalating burden of age-related chronic diseases, such as diabetes, hypertension, and dyslipidemia.

Biological and social constructs, serving as modifiers of health and disease, contribute to the distinction observed between women and men. Substantial individual variability is observed in the aging process between the two sexes [7, 8]. Accumulating evidence [9, 10] also suggests gender disparities in the prevalence and incidence of chronic diseases, such as cardiometabolic diseases. Despite extensive study of gender differences in recent years, there is an overlooked possibility that sex disparities are not static but differ across age groups [10]. For example, the prevalence of diabetes is generally higher in men than in women, but this trend reverses among those aged 75–79 years [11]. Therefore, recognizing the interplay of sex and age is crucial for understanding the trajectories of metabolic diseases and promoting personalized strategies in prevention and management.

To date, only a limited number of studies have examined age-related sex differences in a comprehensive list of risk factors. The Prospective Urban Rural Epidemiological (PURE) study has reported several established metabolic risk factors by age category in both women and men [12]. Additionally, the LifeLines Cohort Study has delved into age-related changes in 51 cardiometabolic risk factors, analyzing the gender differences throughout the lifespan [13]. However, the available evidence is mainly derived from the global or European population, and there is a paucity of gender-specific analyses pertaining to the trajectories of risk factors for adults in China. Meanwhile, menopause is a critical reproductive aging event that indicates the end of fertility in women and is associated with acceleration of cardiovascular disease risk [14, 15]. Whether and when menopause contributes to the heightened cardiovascular risk factors in women still needs to be illustrated.

Our study aims to fill this knowledge gap by comprehensively describing the gender difference in age-specific trajectories of cardiometabolic diseases and their upstream risk factors among Chinese adults. The findings from this study will be particularly valuable for improving metabolic health management and formulating policies aimed at promoting overall health in Chinese population.

## Results

### Project design and summary of the study

A total of 254,670 adults aged 40–80 years from the Risk Evaluation of cAncers in Chinese diabeTic Individuals: a lONgitudinal (REACTION) study were included in the current analysis. Among them, 61,951 (25.2%) had T2DM, 112,788 (44.6%) had hypertension, and 99,574 (39.3%) had dyslipidemia. A total of 175,562 participants (70.3%) had at least one of these metabolic diseases.

**Figure 1:**
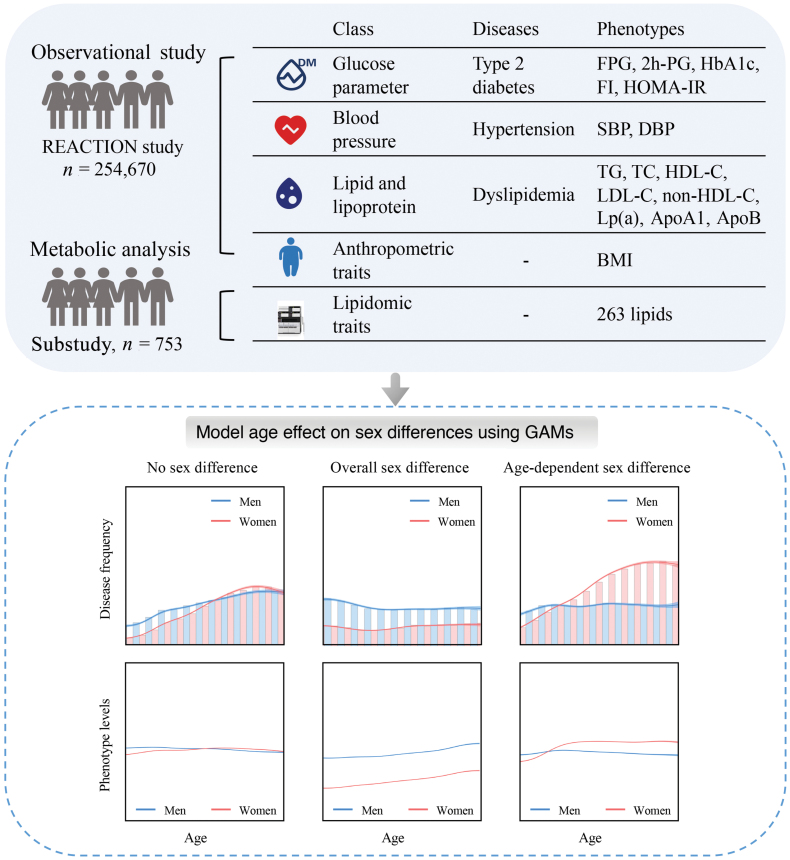
Overview of the REACTION study. In the Risk Evaluation of Cancers in Chinese Diabetic Individuals: a Longitudinal (REACTION) Study, 254,670 adults aged 40−80 years were recruited from resident registration systems from 25 communities across Chinese mainland during 2011−2012, including 166,650 women and 88,020 men. The study collected a total of 16 phenotypic parameters through clinical measurements: FPG, 2h-PG, HbA1c, fasting insulin, HOMA-IR, SBP, DBP, TG, TC, HDL-C, LDL-C, non-HDL-C, LP(a), ApoA1, ApoB, and BMI. Summary statistics for all factors are shown in Supplementary Table S1. GAMs, generalized additive models.

Here, we analyzed the following metabolic risk factors: fasting plasma glucose (FPG), 2-h post-load plasma glucose (2h-PG), hemoglobin A1c (HbA1c), fasting insulin, homeostasis model assessment of insulin resistance (HOMA-IR), diabetes, systolic and diastolic blood pressure (SBP/DBP), hypertension, triglyceride (TG), TC, high-density lipoprotein cholesterol (HDL-C), low-density lipoprotein cholesterol (LDL-C), non-HDL cholesterol (non-HDL-C), Lipoprotein(a) (Lp(a)), apolipoprotein A1 (ApoA1), apolipoprotein B (ApoB), dyslipidemia, and body mass index (BMI) (Fig. 1; Supplementary Table S1). Furthermore, lipidomic data was collected for 753 individuals from six communities of the REACTION study [16].

### Sex disparities in metabolic disease risks across age groups

**Figure 2:**
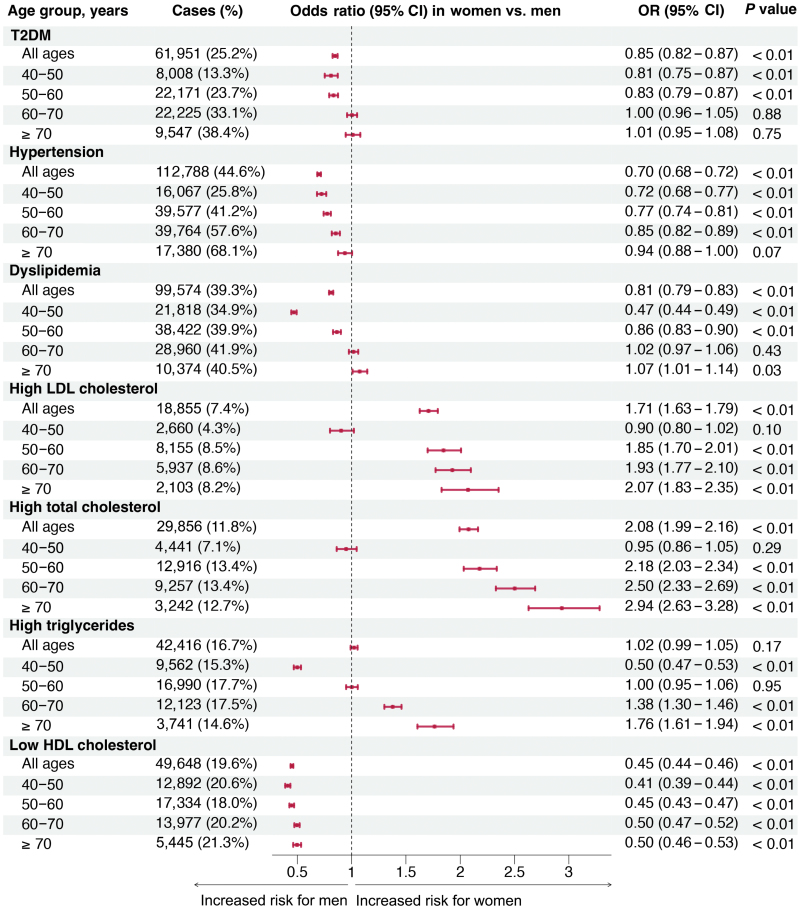
Sex disparities in metabolic disease risks across age groups. Separate adjusted models were performed within each age stratum to find the age-stratum-specific OR values for women versus men, and within each age stratum, the reference group was male. Multivariable models were adjusted for BMI, lifestyle factors (physical activity, smoking, and drinking status), lipid parameters (LDL-C, HDL-C, and TG), FPG, and SBP, but outcome-related risk factors were not included. Thus, FPG for T2DM, SBP for hypertension, and lipid parameters for dyslipidemia were not adjusted.

Figure 2 reports the metabolic disease risks in women versus men stratified by age. In all age groups, women exhibited a lower risk of T2DM, hypertension, and dyslipidemia than men in the general Chinese population. Upon conducting a stratified analysis across four age groups (40–50 years, 50–60 years, 60–70 years, and ≥ 70 years), we revealed that sex differences in dyslipidemia risks were dynamic and changed with age. The age-specific odds ratio (OR) values for dyslipidemia in women, with men as the reference, were as follows: 40–50 years, 0.47 (95% confidence interval (CI), 0.44−0.49); 50–60 years, 0.86 (95% CI, 0.83−0.90); 60–70 years, 1.02 (95% CI, 0.97−1.06); and ≥ 70 years, 1.07 (95% CI, 1.01−1.14). Younger women had a lower risk of dyslipidemia overall and in subtypes (high LDL-C, TC, or TG) than men of a similar age, and these sex differences decreased or even reversed with age advancing. Meanwhile, we found that female participants had lower risks of hypertension and T2DM than male participants across midlife, although differences between the two sexes decreased as they aged and became similar in later life.

### Age-related disparities in metabolic phenotypes by gender

**Figure 3:**
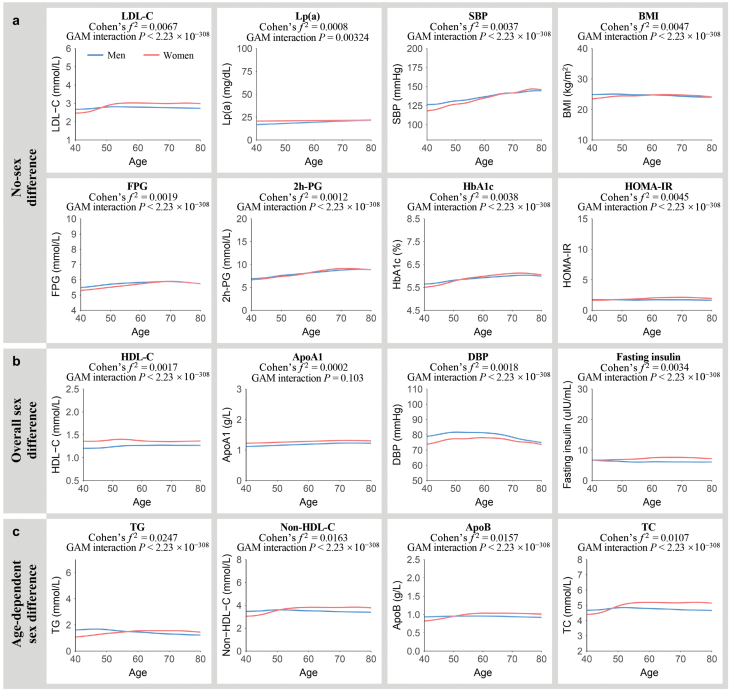
Age-related changes in metabolic risk factors in men and women. (a) Metabolic risk factors with a small effect size for sex differences estimated over the entire age span and age-dependent sex differences (Cohen’s *f*^^2^^_sex/inter_ < 0.01). (b) Metabolic risk factors showing sex differences estimated over the entire age span (Cohen’s *f*^2^_sex_ ≥ 0.01) with no age-dependent sex differences (Cohen’s *f*^2^_inter_ < 0.01). (c) Metabolic risk factors exhibiting age-dependent sex differences (Cohen’s *f*^2^_inter_ ≥ 0.01). Lines correspond to the fitted GAMs. CIs (± 1.96 SE) are plotted around the lines in a more transparent hue. Cohen’s *f*^2^ reflects the effect size of the age-by-sex interaction. GAM interaction *P* represents the significance of the age-by-sex interaction term.

The age-related trajectories of 16 metabolism-related phenotypes, stratified by sex, are illustrated in Fig. 3. Using a generalized additive model (GAM) approach, we found that LDL-C, Lp(a), SBP, BMI, and four blood glucose parameters exhibited no gender differences (Fig. 3a). The levels tended to remain stable across the overall age of the studied population and were comparable between women and men, while several phenotypes exhibited pronounced sex differences across the midlife (*P*_sex_adj_ < 0.05 and Cohen’s *f*^2^_sex_ ≥ 0.01). Women tended to have lower levels of DBP, but higher concentrations of HDL-C, ApoA1, and fasting insulin than men (Fig. 3b).

Our focus extended to phenotypes where sex differences were not static but showed an age-by-sex interaction. We found that four lipid traits demonstrated a significant age-by-sex interaction with considerable effect sizes (*P*_inter_adj_ < 0.05 and Cohen’s *f*^2^_inter_ ≥ 0.01) (Fig. 3c). Women had lower TG, non-HDL-C, ApoB, and TC levels than men until the age of 50−60 years, after which elderly women exhibited higher levels than men. The effect sizes of age-by-sex interaction term for non-HDL-C (Cohen’s *f*^2^ = 0.0163), TC (Cohen’s *f*^2^ = 0.0107), and ApoB (Cohen’s *f*^2^ = 0.0157) were weaker compared to that of TG (Cohen’s *f*^2^ = 0.0247). Notably, even after further correction for metabolic covariates, the age-by-sex interaction effects on TG, non-HDL-C, and ApoB remained statistically significant (Supplementary Table S2).

Furthermore, we conducted a sensitivity analysis to mitigate the potential influence of medication treatment on the levels of metabolic parameters. After excluding participants receiving glucose-lowering, lipid-lowering, or antihypertensive therapy, it demonstrated similar trends with the main analysis (Supplementary Fig. S1; Supplementary Table S3).

### Menopause is associated with age-related sex differences in clinical blood lipids

Next, we zoomed in on the lipid traits with age-dependent sex differences. There was a distinct age-related divergence in lipid levels in women, commencing around 45 years and diminishing by the age of 55. In contrast, men exhibited a relative linear association between age and phenotype features in midlife (Fig. 3c). We further performed exploratory analysis to investigate the effect of menopause status on the observed age-dependent sex differences in clinical blood lipids and aimed to search for the turning point of aging when a strong difference in phenotype levels before and after was observed.

**Figure 4:**
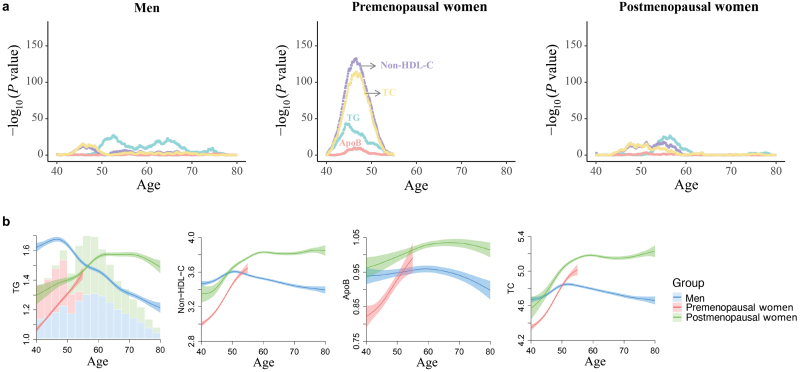
Association of menopause status with blood lipid levels. (a) Age of the most significant differences in lipid levels for different menopause statuses as determined by a sliding window *t*-test. Different lipids are presented in distinct colors. For each phenotype for each year of age, dots represent −log_10_ of *P* value by the *t-*test comparing mean lipid levels before versus after this age. (b) Age-related trajectories of lipid traits among different menopause status. Lines are correspond to the fitted GAMs. CIs (± 1.96 SE) are plotted around the lines in a more transparent hue. In the left panel, bars plot with total counts for men, premenopausal women, and postmenopausal women.

Using a sliding window *t*-test approach, with increment dates by five years, to pinpoint peaks of differentially presented features along the aging trajectories, we found that the sharp differences in lipid levels occurred in premenopausal women before and after the age of 40−50 years, while there were fewer age-related differences in men and postmenopausal women (Fig. 4a). Moreover, no lipid traits showed a significant age-by-sex interaction when excluding the participants around the menopause-related period (45−55 years old) (Supplementary Table S4).

To further understand the importance of menopause transition to age-related sex differences in clinical blood lipids, we presented the age trajectories of lipid levels according to three groups: men, premenopausal women, and postmenopausal women (Fig. 4b). Postmenopausal women had higher lipid levels than premenopausal women, indicating that reproductive axis aging is associated with the worsening in lipid profiles. While, postmenopausal women had lower levels than men at younger ages, after which elderly women exhibited higher levels than men, which was beyond the effects of menopause alone. Therefore, menopause plays an important but not complete role in age-related sex differences in clinical blood lipids.

### Sleep duration affects lipid levels in an age- and sex-dependent way

We further examined the hypothesis that aging trajectories might be slowed down by external intervention strategies. We investigated whether lifestyle factors (current smoking, current drinking, degree of physical activity, status of diet score, or sleep duration per night) would affect the TG and non-HDL-C levels, both showing a large effect size of age-by-sex interaction, in an age- and sex-dependent manner. To minimize external influence on absolute blood lipid levels, we specifically removed participants receiving lipid-lowering medication.

**Figure 5:**
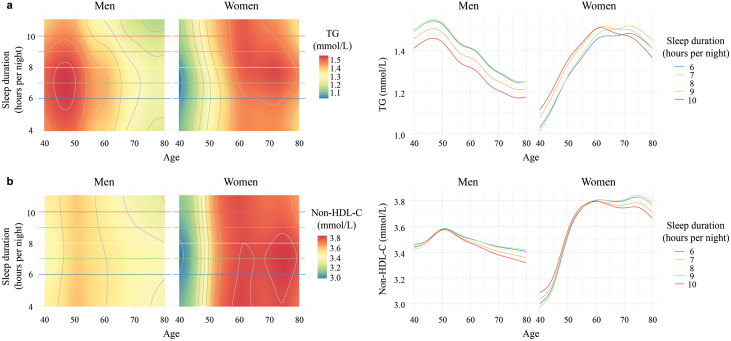
Sleep duration affects lipid levels in an age- and sex-dependent way. The age-dependent effects of sleep duration on TG (a) and non-HDL-C (b) levels in men and women are illustrated through two types of plots. On the left, rasterized heatmap and contour plots depict sleep duration levels (plotted from the 1st to the 99th percentile) on the y-axis, age on the x-axis, and color reflecting lipid levels. The redder the color, the higher the lipid levels. Colored horizontal lines correspond to five values of sleep duration ranging from the 5th to the 95th percentile. The plots on the right show the relationship of lipid (y-axis) with age (x-axis) at five levels of sleep duration ranging from the 5th to the 95th percentile. TG, triglyceride; non-HDL-C, non-high-density lipoprotein cholesterol.

In the stepwise regression analyses, sleep duration emerged as the sole factor exhibiting three-way interactions with both age and sex for lipid levels, showcasing how its effect was modified by the profound sex and age differences. The mean levels of sleep duration were approximately 8 h in both sexes. Men and older women with longer sleep duration (9 or 10 h) showed lower TG levels compared to those of similar age who slept for 6 or 7 h (Fig. 5a). Among younger women, TG levels were lower in those with shorter sleep duration (6 or 7 h), increasing with longer sleep duration (Fig. 5a). As age advanced, non-HDL-C levels tended to be slightly higher in those with shorter sleep duration (Fig. 5b). Based on age- and sex-specific results, longer sleep duration (9 or 10 h) is recommended for men and older women, while shorter (6 or 7 h) is suggested for younger women to achieve a favorable lipid status.

### Age-dependent sex disparities in circulating lipidome

**Figure 6:**
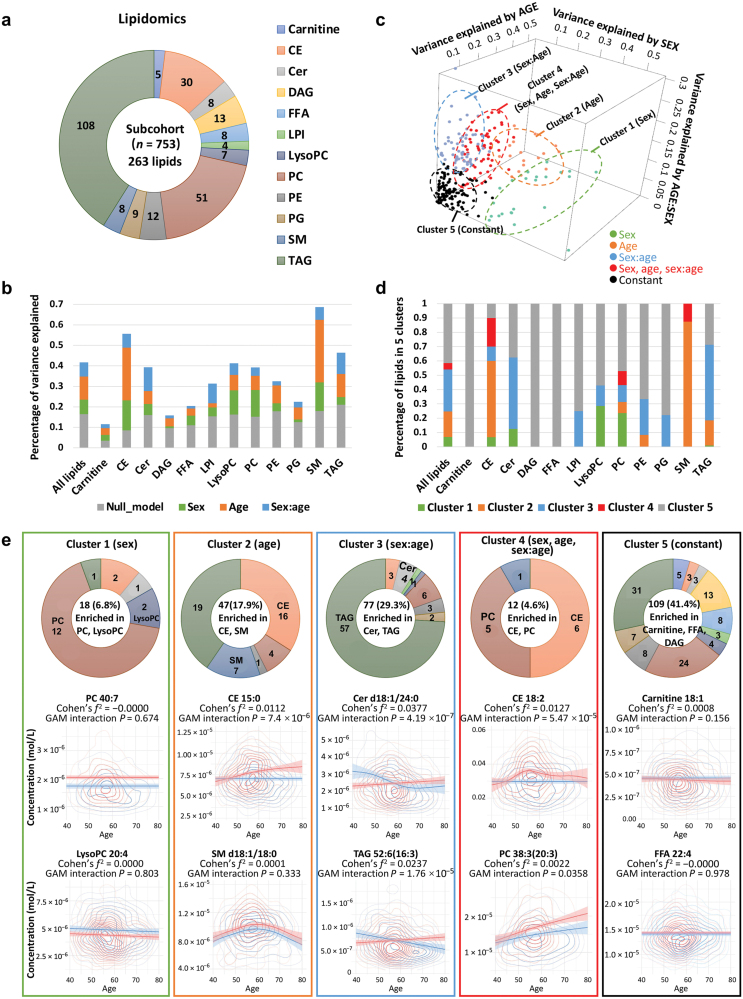
Clustering of age- and sex-specific patterns in lipidomics. (a) Serum lipids detected by HPLC-MRM, comprised 263 lipids across 12 lipid classes. (b) Percentage of variance in serum lipids explained by sex, age, age–sex interaction, and concomitant variables. (c) Clustering of lipids based on patterns of explained variance. (d) Percentage distribution of lipids within each cluster. (e) Enrichment analysis and sex/age-dependent trajectories of lipids in each cluster. The number of lipids in each cluster is summarized in the donut chart.

As a result of the aforementioned findings of substantial impact of age-related sex differences on dyslipidemia risk, cholesterol, and TG levels, we further analyzed lipidomic data to unravel the underlying metabolic disorders and biological mechanisms that contribute to developing specific strategies for early detection and prevention. We investigated lipidomic profile in serum using high-performance liquid chromatography coupled with multiple reaction monitoring (HPLC-MRM), measuring 263 lipids in a subset of 753 participants. This comprehensive analysis encompassed 12 lipid classes, including triacylglycerols (TAGs) and diacylglycerols (DAGs), phosphatidylcholines (PCs), phosphatidylethanolamines (PEs), and so on (Fig. 6a). Strong age-by-sex interaction effects were observed for 30 lipids (11.4%) (Supplementary Fig. S2; Supplementary Table S5). Furthermore, we calculated the impact of sex, age, and their interaction on lipid variance (Fig. 6b; Supplementary Fig. S3; Supplementary Table S6). For all lipids, the combined effect of sex, age, and their interaction accounted for a substantial 25.3% of the variance, which was much stronger than the null model (16.4% of total variance explained by BMI, FPG, and SBP). Meanwhile, sphingomyelins (SMs), cholesteryl esters (CEs), and TAGs were the lipids most strongly impacted by the total effect of sex, age, and their interaction (50.8%, 47.1%, and 25.3% of the total variance, respectively). The average percentages of lipid variance explained by sex, age, and their interaction were 7.1%, 11.2%, and 7.0%, respectively, underscoring the dominant role of age in sharping serum lipid profile. These percentages varied significantly among different lipid classes, with age explaining the most variance of SMs (30%) and the least for lysophosphatidylinositols (LPIs) (2.1%). The effect of sex was weaker than that of age, explaining the most variance for CEs (14.6%) and the least for DAGs (0.9%). Sex-by-age interaction explained slightly less variation than sex, with the highest contribution to variance in ceramides (Cers) (11.7%) and the lowest in free fatty acids (FFAs) (1.2%).

To explore the patterns of age-related sex differences in lipids, five clusters were generated by K-means clustering using specific centers, including sex-related, age-related, sex-by-age interaction related, sex + age + sex-by-age interaction related, and constant (Fig. 6c). Of the 263 lipids, 109 (41.4%) belonged to cluster 5, the largest group, where lipid levels remained constant in both men and women aged 40−80 years, including all 5 carnitines, all 13 DAGs, all 8 FFAs, 7 out of 9 phosphatidylglycerols (PGs), and 8 out of 12 PEs (Fig. 6d). Among the non-constant lipids, 18 (6.8%) were related to sex, 47 (17.9%) were related to age, 77 (29.3%) were related to sex-by-age interaction, and 12 (4.6%) were highly related to all of them (sex + age + sex-by-age interaction). PCs and lysoalkylphosphatidylcholines (LPCs) were enriched in cluster 1, showing distinguishable sex differences but less age-dependent effect. CEs and SMs were enriched in cluster 2, showcasing dynamic changes with age. Cluster 3, the second largest cluster with 77 lipids, displayed age-dependent sex differences, including Cers and TAGs. CEs were enriched in cluster 4 with the highest total effect of sex, age, and sex-by-age interaction. Remarkably, although most CEs were gathered in both cluster 2 (related to age) and cluster 4 (related to sex + age + sex-by-age interaction), as precursors of steroid hormones, five CEs (CE-18:3, CE-22:5, CE-22:6, CE-20:5, CE-18:2, and CE-22:4) were highly affected by sex as well, with contributions from sex of 43.9%, 35.2%, 31.5%, 29.1%, 29.0%, and 24.8%, respectively (Supplementary Table S6). PCs were the other lipid class enriched in cluster 4. It was well known that PCs play essential roles in cell membrane integrity and function, and they were observed to be significantly enhanced with age in both males and females (Fig. 6e).

## Discussion

In this nationwide cohort study including 254,670 participants, we conducted a systematic exploration of the impact of age on sex disparities across established metabolic risk factors and demonstrated dyslipidemia risk and the levels of TG, non-HDL-C, TC, and ApoB with significant age-related sex differences. Throughout the aging process, lipid levels exhibited a more pronounced shift around the age of 40−50 years in premenopausal women, contrasting with a relatively linear association in men and postmenopausal women across the midlife and elderly stages. Menopause exhibited an important but not complete role in age-related sex differences in clinical blood lipids. Meanwhile, the influence of sleep duration on lipid levels also displayed a nuanced modification contingent on both age and gender. Furthermore, lipidomic data demonstrated SMs, CEs, and TAGs as the lipid species most profoundly influenced by the total effect of age, sex, and their interaction. To the best of our knowledge, this study is the first to incorporate age in the evaluation of sex differences across modifiable metabolic factors in large-scale Chinese populations. Our findings underscore the importance of considering age when examining sex differences, emphasizing the need for personalized approaches in the effective prevention and control of metabolic diseases in China.

Sex differences in clinical phenotypes are widely recognized, and while numerous studies have been conducted on this topic, many overlook the crucial covariate of age. Instead, these studies frequently categorize individuals into various age groups [12, 17], which hinders the identification of the specific age associated with phenotype changes. Our research underscores the importance of incorporating age into the analysis of sex differences, as failing to do so can lead to misleading conclusions. To the best of our knowledge, this study is the first to incorporate age in the evaluation of sex differences across modifiable metabolic factors in large-scale Chinese populations. We observed that TG, non-HDL-C, TC, and ApoB demonstrated a significant age-by-sex interaction, with three of the four phenotypes (75.0%) exhibiting no sex differences across the entire age of the studied population. Furthermore, our results showed a decline in sex differences in metabolic disease risks with advancing age, eventually reaching a point of similarity between the two sexes or even reversing, aligning with previous findings [10, 11, 18]. These findings collectively implied that neglecting to consider age could potentially lead to the overlooking of actual sex influences on study outcomes or the drawing of erroneous conclusions. Similar to our observations, the Dutch population cohort Lifelines [13] reported that the levels of several metabolic factors do not show significant sex differences across the entire age span but do manifest sex differences in different ages. Actually, differences between women and men primarily arise from both biological constructs, including sex influences at genetic, molecular, cellular, hormonal, and physiological levels, and social constructs, such as sociocultural factors [19]. Physiological levels of sex hormones undergo significant variations throughout the reproductive and menstrual cycles in women, influencing physiological functions and disease susceptibility across the lifespan [20]. Identifying and understanding the impact of age on gender differences is the first step in establishing age-dependent, gender-specific treatment guidelines where warranted.

In the context of established metabolic risk factors, we observed that in younger age (i.e., younger than 55 years), men consistently exhibited a higher level of DBP and lower levels of HDL-C, ApoA, and fasting insulin compared to women. This trend persisted across the lifespan, which is consistent with previous investigations, such as the INTERHEART study, which reported a higher burden of risk factors in men compared to women at younger age (i.e., < 60 years) [21]. These findings support the notion that efforts to control these risk factors should be initiated at an even younger age in men than in women.

However, we found that as individuals aged beyond 55 years, a contrasting distribution pattern compared to younger participants occurred, with women displaying higher levels of blood TG, non-HDL-C, ApoB, and TC compared to men, consistent with previous studies [22–24]. Notably, women demonstrated a substantial augmentation of age-related increase, particularly around menopausal transition, resulting in a curve-linear rise in lipid levels and a crossing of male age trajectories between the age of 50−60 years. Similar observations were reported in the Prediction for Atherosclerotic Cardiovascular Disease Risk in China (China-PAR) study, where an inverse V-shaped association between age and estimated annual changes of TG, TC, and LDL-C was noted among female participants, with the most significant lipid changes observed in the age group of 40–49 years [25]. When we excluded participants around the menopause period, no lipid traits showed age-by-sex interaction, suggesting that the mechanisms underlying these phenotypes are potentially dependent on menopause and the associated hormonal changes. Midlife is a unique time of life for women that encompasses chronologic aging, reproductive axis aging, and significant life events. Lipid level changes during menopause transition are not just the effects of menopause. When we split the population into three groups: men, premenopausal women, and postmenopausal women, there remained a crossing of age trajectories between postmenopausal women and men in clinical blood lipids. Together, menopause has a profound but not total effect on lipids, and the exact mechanism behind this change remains to be explored.

Besides, lipid management strategies typically target individuals with currently high levels, as the challenge of reversing deleterious cardiovascular effects arises once dyslipidemia emerges. We underscore the necessity to closely monitor the lipid profiles of premenopausal and perimenopausal women, and the critical importance of emphasizing proven lifestyle measures and therapeutic interventions for women in the critical age window of 45−55 years when lipid levels begin to deteriorate and exhibit a greater increasing rate than that in men. Whether the threshold levels for lipid-lowering therapy should change around the time of menopause, or whether the absolute or relative degree of change in lipids (independent of premenopausal levels) predicts future disease events merits further study.

Previous studies have indicated a potential link between sleep duration and blood lipid levels. Our investigation extended this understanding by revealing age- and sex-dependent outcomes, suggesting that longer sleep duration in men and shorter sleep duration in younger women are associated with lower TG levels. In support of our findings, Du *et al.* also observed a higher prevalence of elevated TG levels in men with less than 7 h of sleep duration, with a decrease noted with longer sleep duration [26]. Additionally, previous research from the National Health and Nutrition Survey demonstrated that mean serum TG level was the lowest in women who slept for 6−7 h, progressively increasing with sleep duration beyond 7 h [27]. Meanwhile, participants with short sleep duration tended to present higher non-HDL-C levels during the aging process in both genders. This aligns with earlier studies indicating that short sleep duration constitutes a risk factor for dyslipidemia among men and women aged between 19 and 86 years old [28]. Further investigation is warranted to clarify the physiological mechanisms underlying the relationship between sleep patterns and blood lipid levels.

Interestingly, in our lipidomic analysis, lipids, a class of compounds with diverse structural and functional properties involved in cell membranes, signal transduction, and bioenergetics, demonstrated distinct associations with age and gender. Lipidomic approaches may offer important insights into the aging process and age-related disease etiologies, considering the complex biochemistry of aging. The concentration of SMs was largely influenced by age, accounting for approximately 30% of the total variance, aligning with prior studies [29, 30]. The Leiden Longevity Study (LLS) further supported these findings, indicating that higher levels of SMs  associated with longevity and healthy aging [31], and highlighted the potential role of SMs as an aging biomarker. Furthermore, we identified that gender played a crucial role in determining plasma PC and LPC levels, which was consistent with the results from the VARIET study involving 800 French volunteers [32] and the Australian Diabetes, Obesity and Lifestyle Study (AusDiab) [33]. Males exhibited higher concentrations of LPC, while females showed higher concentrations of PC, indicating sexual dimorphism in human lipidome. Notably, age and gender interactions were most prevalent in TAG profiles. Compared with females, males showed a stronger negative association with age in TAG levels, suggesting that age has a differential effect based on sex. This aligns with previous findings in the Genetics of Lipid-Lowering Drugs and Diet Network (GOLDN) study (where the *P* value of the age–sex interaction term for TG was 0.028) [34]. The age-dependent sex differences in the lipid profiles suggest that there could be a differential impact of aging on lipid metabolism in women compared to men. Understanding these intricate interactions will help identify age- and sex-specific lipidomic biomarkers for personalized interventions.

The main strength of this study lies in the utilization of representative nationwide data, with detailed cardiometabolic phenotypes defined by centralized biochemical measurements and detailed questionnaires. The large sample size allowed us to examine age-related sex differences of modifiable risk factors in Chinese adults for the first time and demonstrate the significance of age consideration in addressing gender disparities in metabolic diseases.

There are several limitations to consider in this study. Firstly, our study focused on individuals aged 40 years and above, encompassing only the midlife and elderly stages, and failing to encompass the entire lifespan. While this age range includes a high-risk period for cardiovascular and metabolic diseases, it also implies that we may have overlooked crucial changes occurring in earlier or later stages of life. Secondly, we employed a cross-sectional design, collecting phenotype data from a cross-sectional cohort, making it challenging to accurately differentiate genuine age effects from those associated with generational influences. To assess age effects more accurately, the use of large-scale longitudinal datasets is warranted to eliminate potential generational influences.

In conclusion, our study provided a comprehensive analysis of modifiable risk factor trajectories across advancing age, employing a sex-stratified approach. We identified three distinct age-related sex difference patterns, with a specific emphasis on blood lipids, and highlighted significant age-by-sex interactions. The aging process exerted varying effects on metabolic disease risks and phenotype levels in women and men. Menopause is associated with age-related sex differences in clinical blood lipids. These findings highlight the important role of age consideration in addressing gender disparities in metabolic diseases and advocate for personalized, age-specific prevention and management. Further research is imperative to explore the mechanisms underlying age-dependent sex differences, aiming to optimize prevention and management efforts for both women and men.

## Materials and methods

### Study design

The REACTION study is a nationwide, multicenter, population-based observational study. The design and methods of the REACTION study have been described previously [35, 36]. Between 2011 and 2012, 259,657 community-dwelling adults aged 40 years and older were recruited from 25 local centers across Chinese mainland. The multicenter study has captured a broad range of data on physical, biochemical, and metabolic functions as well as health status and lifestyle information from the general population. The study protocol and informed consent were approved by the Medical Ethics Committee of Ruijin Hospital affiliated to Shanghai Jiao Tong University School of Medicine, Shanghai, China. All participants signed the written informed consent.

In this study, we included individuals with available sex and age information and focused on the age range from 40 to 80 years due to a limited sample size outside this range. This yielded data for 254,670 individuals, 65.4% of which were females. For these samples, we examined 16 phenotypes including FPG, 2h-PG, HbA1c, fasting insulin, HOMA-IR, SBP, DBP, TG, TC, HDL-C, LDL-C, non-HDL-C, Lp(a), ApoA1, ApoB, and BMI (Supplementary Table S1).

### Data collection

Trained study personnel used standardized questionnaires to collect information on demographic characteristics (age and gender), lifestyle risk factors (including current smoking, current alcohol drinking, physical activity level, diet score, and nighttime sleep duration), and medical history by personal interview. Current smoking was defined as having smoked at least one cigarette per day for the past six months. Current drinking was defined as drinking alcohol at least once a week for the past six months. The International Physical Activity Questionnaire was used to assess leisure-time physical activity during the previous week, and the metabolic equivalent (MET) was calculated to evaluate average weekly energy expenditure [37]. Physical activity was categorized as moderate-to-vigorous (≥ 150 min/week moderate and vigorous intensity or ≥ 600 MET-min per week) and mild (0−600 MET-min per week and 0−150 min/week moderate and vigorous intensity) [38]. Diet score was calculated according to recommendations from the American Heart Association, though whole grain consumption was replaced with bean consumption [39, 40]. It was calculated as the sum of each of the following components: fruits and vegetables ≥ 4.5 cups/day (1 point); fish ≥ 198 g/week (1 point); sweets/sugar-sweetened beverages ≤ 450 kcal/week (1 point); and soy protein ≥ 25 g/day (1 point). A diet score of < 3 represents an unhealthy diet [41, 42]. Sleep duration was recorded as the number of hours slept every 24 h during the previous week.

Each study participant received measurements of height and body weight from the trained staff. BMI was calculated as body weight in kilograms divided by the square of height in meters. Three readings of SBP and DBP obtained by an automated electronic device (OMRON Model HEM-725 FUZZY, Omron Company, Dalian, China) in a seated position after at least a 5-min sitting rest were averaged for analysis.

Blood samples were collected after overnight fasting of at least 10 h and shipped by air in dry ice to the central laboratory of the study located at Shanghai Institute of Endocrine and Metabolic Diseases, which is certified by the College of American Pathologists. The participants undertook a standard 75 g oral glucose tolerance test and post-load blood samples were collected at 2 h. Participants underwent measurements for glycemic measures (FPG, 2h-PG, HbA1c, and fasting insulin), and lipid parameters (TG, TC, LDL-C, HDL-C, Lp(a), ApoA1, and ApoB). HOMA-IR was calculated as FPG (mmol/L) × fasting insulin (mIU/L)/22.5. Non-HDL-C level can be calculated as TC level subtracting HDL-C level. FPG and 2h-PG concentrations were evaluated using the glucose oxidase or hexokinase method under a stringent quality control mechanism. The level of HbA1c was determined by using the method of high-performance liquid chromatography (VARIANTTM Ⅱ and D-10TM Systems, BIO-RAD, Hercules, CA, the United States). Serum insulin and blood lipids were tested using an autoanalyzer (ARCHITECT ci16200, Abbott Laboratories, Abbott Park, IL, the United States) at the central laboratory.

Menopause included those who underwent natural or surgically induced menopause. Women who answered the following question “Do you still have menstruation?” with “Yes” were categorized as premenopausal women. Postmenopausal women were defined as lack of menstrual bleeding for at least 12 months, and the age of menopause was defined as the age at last menstruation. Age at menopause was collected using an interviewer-administered questionnaire. Participants were asked an open-ended question: “At what age did your menstrual period stop?”

### Lipidomic analysis

A total of 263 lipid species (of a targeted library screening > 800 lipids) spanning 12 individual lipid classes were identified and quantitated in 753 participants. Serum lipid profiles were measured by a high-coverage targeted lipidomics approach constructed principally on HPLC-MRM. Lipids were extracted from serum (20 µL) using a modified Bligh and Dyer extraction procedure (double rounds of extraction) and dried in the SpeedVac under OH mode. All lipidomic analyses were performed on an Exion LC system coupled with a QTRAP 6500 PLUS system (Sciex), and individual lipids from various classes were quantitated relative to their respective internal standards, as described previously [16].

### Definition of metabolic diseases

Diabetes was defined as FPG ≥ 7.0 mmol/L, or 2h-PG ≥ 11.1 mmol/L, or HbA1c ≥ 6.5% (48 mmol/mol), or taking antidiabetic medications, or a self-reported previous diagnosis of diabetes by healthcare professional [43]. Hypertension was defined as a SBP ≥ 140 mmHg, or DBP ≥ 90 mmHg, or taking antihypertensive medications. According to the National Cholesterol Education Program (NCEP) Adult Treatment Panel Ⅲ criteria [44, 45], participants with one of the following items were defined as dyslipidemia: TC ≥ 6.22 mmol/L, or TG ≥ 2.26 mmol/L, or HDL-C < 1.04 mmol/L, or LDL-C ≥ 4.14 mmol/L.

### Statistical analysis

For all phenotypes, we removed extreme outliers that were observations of more than three interquartile ranges (IQRs) below the first quartile or more than three IQRs above the third quartile from the whole dataset of phenotypes. Besides, the levels of phenotypes with skewed distribution were log-transformed before analysis. The characteristics of all phenotypes were presented as means (standard deviations) and median in Supplementary Table S1.

Logistic regression models were used to calculate the OR values for metabolic diseases for women compared with men (reference group) in the overall population and four age intervals (40−50 years, 50−60 years, 60−70 years, and ≥ 70 years). Models were adjusted for BMI, lifestyle factors (physical activity, smoking, and drinking status), lipid parameters (LDL-C, HDL-C, and TG), FPG, and SBP, and the outcome-related risk factors were not included in the models. Thus, we did not adjust FPG for T2DM, SBP for hypertension, and lipid parameters for dyslipidemia.

Two methods were used to evaluate age-related sex differences in phenotype levels: age-dependent nonlinear sex differences by fitting a GAM, and general sex differences in the entire age using ordinary least squares linear modeling in R.

#### Using GAMs to examine age-dependent sex differences

Smooth trajectories of phenotype levels by age for men and women were calculated to intuitively study age-dependent nonlinear sex differences by fitting a GAM of the following formula: Phenotype ∼ GAM (sex + s (age) + s (age, by = sex)), where s (age) denotes a spline smooth of age and s (age, by = sex) is an interaction term with smoothing for age-by-sex interaction. Meanwhile, GAM without interaction term was fit for each phenotype to obtain *P* value for the sex difference. For all phenotypes, smoothness selection was done using restricted maximum likelihood (REML) in R using the *mgcv* package [46]. Gaussian family was applied for all phenotypes. *P* values of interaction term were corrected for multiple testing corrections by using Bonferroni correction. To select the phenotypes with a considerable age-by-sex interaction, we further estimated the effect size of the interaction term by calculating Cohen’s *f*^2^ using the following form: *f*^2^ = (*R*_full_^2^−*R*_sub_^2^)/(1 − *R*_full_^2^), where *R*_full_^2^ and *R*_sub_^2^ were the proportion of variance explained (PVE) by the model with/without an age × sex (ordered) interaction term, respectively [47]. Overall, we considered the phenotypes to show age-by-sex interaction, if *P* value of the age–sex interaction term in the GAM was significant after Bonferroni correction for multiple testing (age-by-sex interaction *P*_inter_adj_ < 0.05) and the effect size of this interaction was considerable (Cohen’s *f*^2^_inter_ ≥ 0.01).

#### Using linear models to examine overall sex differences

Besides, we assessed the general linear sex differences over the life course when not taking age into account by using ordinary least squares linear modeling. Similarly, we considered the phenotypes to show an overall sex difference, if *P* value of the sex term was significant after Bonferroni correction for multiple testing (*P*_sex_adj_ < 0.05) and the effect size was considerable (Cohen’s *f*^2^_sex_ ≥ 0.01).

#### Analysis to estimate age-by-sex interactions associated with menopause

To further confirm the accurate age of phenotype changes in women and men, and detect whether a different age-related divergence in lipid levels occurred in three menopause statuses, we conducted a slide window analysis in which a *t*-test was performed for each year of age, comparing mean phenotype levels in a window of five years before versus five years after that age. Results were presented as −log_10_(*P* value).

To check the effect of age on sex differences excluding the menopause period, we split our cohort into two groups: before menopause (age < 45 years) and after menopause (age > 55 years), and ran the primary analysis (GAM with age, sex, and age-by-sex interaction terms) in these two groups separately, calculating whether phenotypes still with a significant age-by-sex interaction (Bonferroni-adjusted *P* < 0.05 and Cohen’s *f*^2^_inter_ ≥ 0.01).

To check whether menopause plays an important role in the age–sex interaction of lipid traits, we used GAMs to fit the age-related trajectories of lipids in three groups: men, premenopausal women, and postmenopausal women.

#### Estimating the effect of lifestyle factors on lipids

We further checked whether lifestyle factors affected the levels of TG and non-HDL-C in an age- and sex-dependent way. In the GAM, TG and non-HDL-C were treated as outcome variables. The predictive factors included age, sex, and five lifestyle factors together with their two-way interactions with age, their two-way interactions with sex, and their three-way interactions with both age and sex. Using the forward stepwise selection algorithm (*P* < 0.05), the lifestyle factors that exhibited three-way interactions with both age and sex were selected into the final GAM model.

#### Calculation of explained variance

To determine whether serum lipidomics were influenced by sex and age and classify lipoproteins based on patterns of age-dependent sex differences and on the age and sex contribution to variance in their levels, we calculated the variance of each lipid explained by the covariates of sex, age and age-by-sex interaction. We fitted four separate GAMs of the following formulas: (i) null model: Lipid level ∼ GAM (BMI + FPG + SBP); (ii) sex-related model: Lipid level ∼ GAM (BMI + FPG + SBP + sex); (iii) age-related model: Lipid level ∼ GAM (BMI + FPG + SBP + sex + s (age)); and (iv) interaction-related model: Lipid level ∼ GAM (BMI + FPG + SBP + sex + s (age) + s (age, by = sex)). *R*^2^ was the PVE by the model. Variance explained by sex, age, and age-by-sex interaction was calculated as *R*^2^ (formula 2) − *R*^2^ (formula 1), *R*^2^ (formula 3) − *R*^2^ (formula 2), *R*^2^ (formula 4) − *R*^2^ (formula 3), respectively. The results are presented in a circular bar plot which was plotted using the R package “circlize” (v.0.4.15). Hierarchical clustering (hclust) was performed using Euclidean distance and the unweighted pair group method with the arithmetic mean (UPGMA) agglomeration method.

#### K-means clustering for lipids

To identify lipid trajectory patterns in men and women over age, K-means clustering was performed on PVE calculated by previous analysis, and all lipids were assigned into five distinct clusters. The five centers were selected as follows: (i) (*x* = max of PVE by sex, *y* = 0, *z* = 0) for sex-dependent cluster; (ii) (*x* = 0, *y* = max of PVE by age, *z* = 0) for age-dependent cluster; (iii) (*x* = 0, *y* = 0, *z* = max of sex:age) for sex:age-dependent cluster; (iv) (*x* = max of PVE by sex, *y* = max of PVE by age, *z* = max of sex:age) for cluster related to the total effect of sex, age, and sex:age interaction; and (v) (*x* = 0, *y* = 0, *z* = 0) for cluster with constant lipid level. Lipid pathway enrichment analysis was performed by Fisher’s exact test for all the five clusters.

#### Sensitivity analysis

For all selected phenotypes, we conducted sensitivity analysis by adding smoking status (yes/no), T2DM (yes/no), and nonlinear effect of BMI, FPG, TC, HDL-C, and SBP to the GAMs. If a covariate was relevant to the phenotype of interest, we excluded it from the models. Thus, we did not correct TC and HDL-C for lipid phenotypes, FPG, and T2DM for blood glucose phenotypes, SBP for blood pressure measurements, and BMI for anthropometric traits.

Besides, these levels were possible to change after the use of antihypertension, antidiabetic, or lipid-lowering medications. Therefore, we further checked the age and sex patterns among those without medication usage.

## Supplementary Material

loae032_suppl_Supplementary_Figures

loae032_suppl_Supplementary_Tables

## Data Availability

The authors confirm that all the data supporting the findings of this study are available within the supplementary material and corresponding authors.

## The 4C Study Group members are as follows:

Steering Committee: Guang Ning, Shanghai National Clinical Research Center for Endocrine and Metabolic Diseases, Key Laboratory for Endocrine and Metabolic Diseases of the National Health Commission of the People’s Republic of China, Shanghai Institute of Endocrine and Metabolic Diseases, Ruijin Hospital, Shanghai Jiao Tong University School of Medicine, Shanghai, China; Yiming Mu, Chinese People’s Liberation Army General Hospital, Beijing, China; Jiajun Zhao, Shandong Provincial Hospital affiliated to Shandong University, Jinan, China; Weiqing Wang, Shanghai National Clinical Research Center for Endocrine and Metabolic Diseases, Key Laboratory for Endocrine and Metabolic Diseases of the National Health Commission of the People’s Republic of China, Shanghai Institute of Endocrine and Metabolic Diseases, Ruijin Hospital, Shanghai Jiao Tong University School of Medicine, Shanghai, China; Chao Liu, Jiangsu Province Hospital on Integration of Chinese and Western Medicine, Nanjing, China; Yufang Bi, Shanghai National Clinical Research Center for Endocrine and Metabolic Diseases, Key Laboratory for Endocrine and Metabolic Diseases of the National Health Commission of the People’s Republic of China, Shanghai Institute of Endocrine and Metabolic Diseases, Ruijin Hospital, Shanghai Jiao Tong University School of Medicine, Shanghai, China; Donghui Li, Department of Gastrointestinal Medical Oncology, The University of Texas MD Anderson Cancer Center, Houston, TX, United States; Shenghan Lai, Johns Hopkins University School of Medicine, Baltimore, MD, United States; Zachary T. Bloomgarden, Icahn School of Medicine at Mount Sinai, New York, United States.

Working Group: Weiqing Wang, Yufang Bi, Jieli Lu, Mian Li, Shanghai National Clinical Research Center for Endocrine and Metabolic Diseases, Key Laboratory for Endocrine and Metabolic Diseases of the National Health Commission of the People’s Republic of China, Shanghai Institute of Endocrine and Metabolic Diseases, Ruijin Hospital, Shanghai Jiao Tong University School of Medicine, Shanghai, China; Yiming Mu, Chinese People’s Liberation Army General Hospital, Beijing, China; Jiajun Zhao, Shandong Provincial Hospital affiliated to Shandong University, Jinan, China; Chao Liu, Jiangsu Province Hospital on Integration of Chinese and Western Medicine, Nanjing, China; Lulu Chen, Union Hospital, Tong ji Medical College, Huazhong University of Science and Technology, Wuhan, China; Lixin Shi, Affiliated Hospital of Guiyang Medical College, Guiyang, China; Qiang Li, The Second Affiliated Hospital of Harbin Medical University, Harbin, China; Tao Yang, The First Affiliated Hospital of Nanjing Medical University, Nanjing, China; Li Yan, Sun Yat-sen Memorial Hospital, Sun Yat-sen University, Guangzhou, China; Qin Wan, The Affiliated Hospital of Luzhou Medical College, Luzhou, China; Shengli Wu, Karamay Municipal People’s Hospital, Xinjiang, China; Guixia Wang, The First Hospital of Jilin University, Changchun, China; Zuojie Luo, Yingfen Qin, The First Affiliated Hospital of Guangxi Medical University, Nanning, China; Xulei Tang, The First Hospital of Lanzhou University, Lanzhou, China; Gang Chen, Fujian Provincial Hospital, Fujian Medical University, Fuzhou, China; Yanan Huo, Jiangxi Provincial People’s Hospital Affiliated to Nanchang University, Nanchang, China; Zhengnan Gao, Dalian Municipal Central Hospital, Dalian, China; Qing Su, Xinhua Hospital Affiliated to Shanghai Jiao Tong University School of Medicine, Shanghai, China; Zhen Ye, Ruying Hu, Zhejiang Provincial Center for Disease Control and Prevention, Zhejiang, China; Youmin Wang, The First Affiliated Hospital of Anhui Medical University, Hefei, China; Guijun Qin, The First Affiliated Hospital of Zhengzhou University, Zhengzhou, China; Huacong Deng, The First Affiliated Hospital of Chongqing Medical University, Chongqing, China; Xuefeng Yu, Tongji Hospital, Tongji Medical College, Huazhong University of Science and Technology, Wuhan, China; Feixia Shen, The First Affiliated Hospital of Wenzhou Medical University, Wenzhou, China; Li Chen, Qilu Hospital of Shandong University, Jinan, China
