# Supplementary material for: Exploring age and gender disparities in cardiometabolic phenotypes and lipidomic signatures among Chinese adults: a nationwide cohort study
Source: Life Metab. 2024 Aug 2;3(5):loae032. doi: 10.1093/lifemeta/loae032 (PMC11749084; doi:10.1093/lifemeta/loae032)
Supplement: loae032_suppl_Supplementary_Figures [file loae032_suppl_Supplementary_Figures.docx]

**Supplementary Figure S1** Age-dependent sex differences for metabolic risk factors among those without medication usage. Lines correspond to the fitted GAMs: the blue line represents men, and the red line represents women. Confidence intervals (± 1.96 SE) are plotted around the lines in a more transparent hue. Cohen’s *f^2^* reflects the effect size of the age-by-sex interaction. GAM interaction *P* represents the significance of the age-by-sex interaction term.

**Supplementary Figure S2** Age-dependent sex differences for serum lipidomic traits. For each phenotype, the plots show the density of observations for men (blue contours) and women (red contours) and fitted lines obtained using GAM: men (blue) and women (red). Confidence intervals (± 1.96 SE) around the lines are depicted in a more transparent hue. Cohen’s *f^2^* reflects the effect size of the age-by-sex interaction. GAM interaction *P* indicates the significance of the age-by-sex interaction term.

**Supplementary Figure S3** Variance in serum lipidomic traits explained by age, sex, and their interaction. The fraction of total variance explained by sex (green), age (orange), and age-by-sex interaction (blue) in addition to covariates (grey) is visualized in the circular bar plot for each lipid. Bar height reflects the explained variance proportion. The inner part of the plot represents hierarchical clustering of age-dependent patterns of sex differences.

**Supplementary Table S1** Characteristics of phenotype traits used in the current study. For each phenotype, basic statistics are given for all samples and stratified by sex: mean, standard deviations (SD), and median are shown.

**Supplementary Table S2** Age-dependent sex differences in general phenotypes based on the Generalized Additive Model (GAM) fitting, with and without covariate adjustment. GAM fitting results including the GAM age-by-sex interaction term *P* value and effect size estimated using Cohen's *f^2^* are provided for each phenotype. Linear sex differences over the whole age span were studied using linear models, where sex was the single predictor; both the sex term significance and effect size are given. Non-linear sex differences over the whole age span were studied using GAMs, where the interaction term was omitted; both the sex term significance and effect size are given. The right part of the table shows similar parameters after adjusting for the major metabolic risk factors: BMI, fasting plasma glucose, total cholesterol, HDL cholesterol, systolic blood pressure, current smoking, and type 2 diabetes.

**Supplementary Table S3** Age-dependent sex differences for metabolic disease risk factors among those without medication usage. GAM fitting results including the GAM age by sex interaction term *P* value and effect size estimated using Cohen's *f^2^* are provided for each phenotype. Linear sex differences over the whole age span were studied using linear models, where sex was the single predictor; both the sex term significance and effect size are given. Non-linear sex differences over the whole age span were studied using GAMs, where the interaction term was omitted; both the sex term significance and effect size are given.

**Supplementary Table S4** Age-dependent sex differences in phenotypes excluding the age of menopause. GAM fitting results are reported for two age groups that exclude the age of menopause: before 45 years old (left table) and after 55 years old (right table). The GAM age by sex interaction term *P* value and effect size estimated using Cohen's *f^2^* are provided for each phenotype.

**Supplementary Table S5** Age-dependent sex differences in serum lipidomic traits based on the GAM fitting. Age-dependent sex differences for each lipidomic trait are provided based on GAM age-by-sex interaction *P* value significance and effect size estimated using Cohen's *f^2^*. Non-linear sex differences over the whole age span were estimated using GAMs, where the interaction term was omitted; the sex term significance and effect size (Cohen's *f^2^*) are provided. The non-linear age effect for both sexes combined was estimated using GAMs, where the interaction term was omitted; the age term significance and effect size (Cohen's *f^2^*) are provided.

**Supplementary Table S6** The proportion of lipid variance is explained by age, sex, and their interaction and K-means clustering for distinguished patterns. The table provides the explained variance (*R^2^*) of each serum lipidomic trait by covariates (null_model), sex, age, and age-by-sex interaction estimated using GAMs. In addition, it shows the position of each lipoprotein in the hierarchical clustering and the cluster group, as presented in Supplementary Figure S3.
