# Supplementary material for: Exploring age and gender disparities in cardiometabolic phenotypes and lipidomic signatures among Chinese adults: a nationwide cohort study
Source: Life Metab. 2024 Aug 2;3(5):loae032. doi: 10.1093/lifemeta/loae032 (PMC11749084; doi:10.1093/lifemeta/loae032)
Supplement: loae032_suppl_Supplementary_Tables [file loae032_suppl_Supplementary_Tables.pdf]

Supplementary Table S1 Characteristics of phenotype traits used in the current study.

Phenotype	Unit	N	N men	N women	mean (sd)	mean (sd) men	mean (sd) women	median	median men	median women	Log trans
FPG	mmol/L	239887	81853	158034	5.684 (0.864)	5.771 (0.910)	5.639 (0.836)	5.5	5.58	5.47	
2h-PG	mmol/L	240082	82238	157844	7.967 (3.101)	8.039 (3.327)	7.929 (2.976)	7.14	7.15	7.13	
HbA1c	%	242891	83049	159842	5.885 (0.589)	5.885 (0.614)	5.885 (0.576)	5.8	5.8	5.8	
Fasting insulin	uIU/mL	232840	80307	152533	7.727 (4.410)	7.124 (4.418)	8.045 (4.372)	6.7	6.1	7	LOG
HOMA-IR		228912	78905	150007	2.081 (1.476)	1.967 (1.497)	2.141 (1.462)	1.697	1.572	1.758	LOG
SBP	mm Hg	252484	87192	165292	133.074 (20.257)	135.446 (19.643)	131.823 (20.463)	131	133.3	129.7	
DBP	mm Hg	252433	87173	165260	78.217 (10.888)	80.404 (10.995)	77.063 (10.652)	77.7	80	76.3	
HDL-C	mmol/L	253644	87613	166031	1.332 (0.348)	1.254 (0.346)	1.373 (0.342)	1.3	1.21	1.35	
LDL-C	mmol/L	253627	87611	166016	2.863 (0.837)	2.775 (0.810)	2.909 (0.847)	2.81	2.73	2.86	
TC	mmol/L	253706	87650	166056	4.937 (1.080)	4.771 (1.045)	5.025 (1.088)	4.91	4.74	5.01	
TG	mmol/L	253452	87505	165947	1.593 (1.035)	1.651 (1.135)	1.562 (0.976)	1.3	1.31	1.3	LOG
Non-HDL-C	mmol/L	253631	87611	166020	3.605 (0.969)	3.516 (0.949)	3.652 (0.976)	3.54	3.45	3.59	
Lp(a)	mg/dL	10125	3854	6271	20.372 (14.574)	19.158 (14.315)	21.119 (14.683)	18	16	19	
ApoA1	g/L	10176	3862	6314	1.255 (0.267)	1.197 (0.263)	1.290 (0.264)	1.22	1.15	1.25	LOG
ApoB	g/L	10172	3867	6305	0.975 (0.232)	0.948 (0.225)	0.992 (0.235)	0.96	0.92	0.97	
BMI	kg/m^2	249335	86057	163278	24.581 (3.364)	24.718 (3.216)	24.508 (3.438)	24.35	24.626	24.204	

For each phenotype basic statistics are given for all samples and stratified by sex: mean, standard deviations (SD) and median.

Supplementary Table S2 Age-dependent sex differences in general phenotypes based on the Generalized Additive Model (GAM) fitting, with and without covariate adjustment.

Phenotype	Basic model						Multivariable model	
	**Interaction_P value_adj_Bonferroni**	**Interaction_effect_Cohen_f²**	**Sex_gam_P value_adj_Bonferroni**	**Sex_gam_effect_Cohen_ f²**	**Sex_lm_P value_adj_Bonferroni**	**Sex_lm_effect_Cohen_ f²**	**Interaction_P value_adj_Bonferroni**	**Interaction_effect_Cohen_ f²**
FPG	0	0.001858162	2.62E-197	0.003770482	6.98E-276	0.005288	0	0.000303629
2h-PG	0	0.001174887	1	-3.84E-06	2.56E-15	0.000283	0	0.000395221
HbA1c	0	0.003789568	5.37E-18	0.000325417	1	5.68E-08	0	0.001453396
Fasting insulin	0	0.00344124	0	0.0195246	0	0.018737	0.178268263	5.79E-05
HOMA-IR	0	0.004491651	0	0.00827442	0	0.007208	0.048657365	6.56E-05
SBP	0	0.00367187	1.12E-222	0.004043474	0	0.007281	0	0.001457655
DBP	0	0.001799331	0	0.023485646	0	0.021742	0	0.000563072
HDL-C	0	0.001651867	0	0.02661062	0	0.027258	0	0.000752822
LDL-C	0	0.006698571	0	0.006477616	0	0.005875	0	0.004878132
TC	0	0.010678115	0	0.01377732	0	0.012764	0	0.008088646
TG	0	0.024679131	2.15E-51	0.000915456	2.50E-55	0.000991	0	0.01651216
Non-HDL-C	0	0.016300977	2.37E-284	0.005148897	3.06E-246	0.00446	0	0.011634591
LP(a)	0.051853263	0.000801794	2.81E-10	0.004375555	7.52E-10	0.004287	0.304347744	0.000470144
ApoA1	1	0.00021328	5.87E-72	0.032515073	3.18E-69	0.031425	1	0.00016253
ApoB	0	0.015687491	1.68E-19	0.008442405	2.13E-19	0.008551	0	0.011420452
BMI	0	0.004726871	3.82E-47	0.00085188	3.34E-48	0.000876	0	0.000745528

GAM fitting results including the GAM age-by-sex interaction term P value and effect size estimated using Cohen's f2 are provided for each phenotype. Linear sex differences over the whole age span were studied using linear models, where sex was the single predictor; both the sex term significance and effect size are given. Non-linear sex differences over the whole age span were studied using GAMs, where the interaction term was omitted; both the sex term significance and effect size are

given. The right part of the table shows similar parameters after adjusting for the major metabolic risk factors: BMI, FPG, TC, HDL-C, SBP, current smoking and T2DM. Relevant covariates were excluded from the models. Specifically, TC and HDL-C were not corrected for lipid phenotypes, FPG and T2DM for blood glucose phenotypes, SBP for blood pressure measurements, and BMI for anthropometric traits.

Supplementary Table S3 Age-dependent sex differences for metabolic disease risk factors among those without medication usage.

Phenotype	Interaction_P value_adj_Bonferroni	Interaction_effect_Cohen_f^2	Sex_gam_P value_adj_Bonferroni	Sex_gam_effect_Cohen_ f^2	Sex_lm_P value_adj_Bonferroni	Sex_lm_effect_Cohen_ f^2	Age_gam_P value_adj_Bonferroni	Age_gam_effect_Cohen_ f^2
FPG	0	0.001562	6.379E-156	0.0036337	3.83E-207	0.004842	0	0.020267
2h_PG	0	0.000692	1.63984E-20	0.0004693	0.713878352	2.09E-05	0	0.03792116
HbA1c	0	0.004942	3.12183E-46	0.0010467	1.51E-10	0.000234	0	0.06481958
Fasting insulin	0	0.003504	0	0.0205572	0.00E+00	0.020608	0	0.0005381
HOMA_IR	0	0.00422	0	0.0094024	0	0.008938	0	0.00231353
SBP	0	0.003242	0	0.0071287	0.00E+00	0.011422	0	0.09873234
DBP	0	0.001442	0	0.0261564	0	0.024806	0	0.00917907
HDL_C	0	0.001789	0	0.0243604	0.00E+00	0.024173	0	0.00279586
TC	0	0.011145	0	0.0102715	0	0.008971	0	0.03226411
LDL_C	0	0.007569	4.2023E-223	0.005002	2.1876E-188	0.004225	0	0.02858871
TG	0	0.024562	3.033E-108	0.0024141	1.62E-110	0.00247	0	0.00639117
Non_HDL_C	0	0.017214	4.5042E-138	0.0030829	2.5719E-108	0.002419	0	0.03292265
Lp(a)	0.04751855	0.000886	8.44613E-11	0.0049296	2.96809E-10	0.004815	4.04723E-06	0.00276151
ApoA1	0.69383139	0.000398	4.90136E-63	0.0306548	2.44541E-60	0.029452	0	0.01217435
ApoB	0	0.015954	6.42436E-16	0.0073726	9.26073E-16	0.007452	0	0.02987885
BMI	0	0.004599	2.21416E-53	0.0012013	5.25889E-48	0.001084	0	0.00294962

GAM fitting results including the GAM age by sex interaction term P value and effect size estimated using Cohen's f2 are provided for each phenotype. Linear sex differences over the whole age span were studied using linear models, where sex was the single predictor; both the sex term significance and effect size are given. Non-linear sex differences over the whole age span were studied using GAMs, where the interaction term was omitted; both the sex term significance and effect size are given.

Supplementary Table S4 Age-dependent sex differences in phenotypes excluding the age of menopause.

Phenotype	Before 45 years old			After 55 years old		
	Interaction_P value	Interaction_P value_adj_Bonferroni	Interaction_effect_Cohen_f^2	Interaction_P value	Interaction_P value_adj_Bonferroni	Interaction_effect_Cohen_f^2
BMI	0	0	0.00069909	0	0	0.001246282
HDL_C	0.20431013	1	3.27486E-05	0	0	0.000422207
LDL_C	0	0	0.000764674	0.0119559	0.1912948	4.87209E-05
TC	0	0	0.00087652	0	0	0.000333991
TG	6.6826E-06	0.000106921	0.000369942	0	0	0.003701086
Non_HDL_C	0	0	0.00088037	0	0	0.000689765
Lp(a)	0.48691898	1	-3.80301E-07	0.9636741	1	2.69964E-06
ApoA1	0.4458695	1	1.43147E-07	0.8044118	1	-3.29125E-07
ApoB	0.80173964	1	6.62748E-10	0.0573711	0.91793682	0.000502033
FPG	0.51650784	1	-1.14984E-07	0	0	0.001182097
2h_PG	0.00049051	0.007848102	0.000198711	0	0	0.000778759
HbA1c	0.07418897	1	6.82253E-05	0	0	0.000312295
Fasting insulin	0.75156375	1	-1.2102E-06	0	0	0.000307332
HOMA_IR	0.02763115	0.442098443	0.000167311	0	0	0.00078429
SBP	0	0	0.000969097	0	0	0.001343671
DBP	0	0	0.000437478	0	0	0.000993557

GAM fitting results are reported for two age groups that exclude the age of menopause: before 45 years old (left table) and after 55 years old (right table). The GAM age by sex interaction term P value and effect size estimated using Cohen's f2 are provided for each phenotype.

Supplementary Table S5 Age-dependent sex differences in serum lipid-omics traits based on GAM fitting.

Lipidomics	Class	Inter_P_adj _Bonferroni	Cohen_f^2	Sex_gam_P_adj_ Bonferroni	Sex_gam_ Cohen_f^2	G_lm_P_adj_ bonferroni	G_lm_ Cohen_f^2	Age_gam_P_adj _Bonferroni	Age_gam_ Cohen_f^2
Cer d18:1/16:0	Cer	0.016819	0.0095592	1	0.006878	1	0.006642	1	0.010989
Cer d18:0/16:0	Cer	1	0.0039422	1	0.002187	1	0.003096	1	-9.23E-05
Cer d18:1/18:0	Cer	0.265944	0.0049339	1	0.008474	1	0.009916	1	0.011027
Cer d18:0/18:0	Cer	1	0.0009809	1	0.000663	1	0.002288	1	-0.000991
Cer d18:1/22:0	Cer	0.004499	0.0259327	1	-0.000174	1	0.001701	1	0.002107
Cer d18:0/22:0	Cer	0.001339	0.0279415	1	0.001725	1	0.004297	1	0.006324
Cer d18:1/24:1	Cer	1	9.29E-05	1	0.006468	1	0.008293	1	0.001742
Cer d18:1/24:0	Cer	0.00011	0.0376808	1	-0.000106	1	0.000837	1	0.001592
FFA22:6	FFA	1	1.13E-06	1	0.000821	1	0.001249	1	0.007784
FFA22:5	FFA	1	-1.84E-07	1	0.005211	1	0.005631	1	0.001705
FFA22:4	FFA	1	-3.03E-06	1	-0.00011	1	0.001241	1	-0.001335
FFA18:2	FFA	1	0.0001946	1	0.003592	1	0.004917	1	-0.001305
FFA18:1	FFA	1	-4.02E-07	1	0.009789	1	0.00969	1	0.002989
FFA18:0	FFA	1	0.0004785	1	0.005314	1	0.009833	1	0.010905
FFA16:1	FFA	1	0.0039717	1	-0.001407	1	4.21E-05	1	0.004236
FFA16:0	FFA	1	-3.85E-06	1	0.001579	1	0.003989	1	0.004632
PG38:6(22:5)	PG	1	0.0009264	1	0.002744	1	0.003324	1	0.001871
PG38:5(22:5)	PG	1	9.37E-07	1	-0.001331	1	1.71E-05	1	-0.000813
PG36:3(18:1)	PG	1	-1.76E-06	1	0.00391	1	0.00713	1	0.0078
PG36:2	PG	1	0.005104	1	0.000748	1	0.00375	1	0.011596
PG36:1	PG	1	0.0010382	1	-9.86E-05	1	0.002433	1	0.00858
PG38:6	PG	1	-3.45E-06	1	-0.001276	1	0.00028	1	0.001396
PG38:5	PG	1	-0.000177	1	-0.001185	1	3.41E-07	1	0.007528

PG38:4	PG	1	-6.61E-06	1	0.000563	1	0.002776	1	0.001507
PG38:3	PG	1	-4.51E-05	1	-0.000792	1	0.00103	1	0.002042
PE32:1	PE	1	0.0003743	1	-0.001052	1	8.66E-05	1	0.003475
PE34:2	PE	1	0.0032091	1	0.000463	1	0.002689	1	0.003918
PE36:3	PE	1	-1.40E-07	1	0.006501	1	0.009827	1	0.004613
PE36:2	PE	1	0.0025621	1	0.006239	1	0.010769	1	0.011702
PE42:3p	PE	1	1.77E-06	1	0.002339	1	0.002188	0.31404	0.012806
PE40:5p	PE	1	4.82E-05	1	0.003937	1	0.00741	1	0.006227
PE38:5p	PE	1	-3.60E-05	1	-0.0013	1	0.000147	0.005926	0.023041
PE38:4p	PE	1	-6.98E-06	1	-0.001264	1	5.69E-05	0.055523	0.017213
PE36:1p	PE	1	0.0040994	1	0.003487	1	0.006232	1	0.004845
PE38:5p(20:4)	PE	1	8.49E-06	1	-0.001282	1	8.23E-05	0.02696	0.019067
PE38:4p(20:4)	PE	1	3.69E-08	1	-0.001122	1	1.16E-06	0.439071	0.01197
PE40:5p(22:5)	PE	1	-7.15E-07	0.8665977	0.010286	0.431274	0.013352	1	0.002232
PC32:2	PC	1	0.0010994	2.65E-08	0.056106	3.34E-08	0.056802	1	-0.000658
PC32:1	PC	1	4.41E-06	1	0.003051	1	0.002082	0.000745	0.029423
PC32:0	PC	1	-5.83E-05	1	0.004354	1	0.003653	0.213939	0.014695
PC34:3	PC	1	0.0072245	2.22E-07	0.05075	4.27E-07	0.050289	1	0.000824
PC36:6	PC	0.000861	0.0271078	1.82E-07	0.050465	9.28E-08	0.054213	1	0.004306
PC36:5	PC	0.002231	0.0241344	1	0.006437	1	0.00759	1	0.001024
PC38:5	PC	1	0.008275	0.0071836	0.021672	0.005356	0.024518	1	-0.000711
PC40:7	PC	1	-1.11E-06	2.96E-05	0.035903	2.06E-05	0.039339	1	-0.000445
PC40:6	PC	1	0.0063129	0.0515827	0.016919	0.05072	0.018718	1	0.003763
PC40:5	PC	1	0.0030582	0.0027547	0.024152	0.003352	0.02588	1	-9.69E-05
PC40:4	PC	1	-1.67E-06	1	0.00451	1	0.005954	1	-0.001323
PC40:3	PC	1	0.002289	1	-0.001036	1	0.000307	1	-0.00053

SM d18:0/18:0	SM	1	0.0001706	1	0.006672	1	0.009613	0.017755	0.032895
SM d18:1/18:0	SM	1	6.91E-05	0.2825149	0.01287	0.103412	0.016882	0.001286	0.041909
SM d18:1/20:0	SM	1	-1.12E-05	1	0.005708	1	0.00953	0.105069	0.026749
SM d18:1/21:0	SM	0.547411	0.0102158	7.74E-06	0.040152	4.04E-06	0.043572	0.018756	0.031786
SM d18:1/22:0	SM	1	0.0056853	1	-0.000558	1	0.001718	1	0.016446
SM d18:1/23:0	SM	0.034179	0.0156784	0.3923957	0.01198	0.229076	0.014878	1	0.018057
SM d18:1/24:0	SM	1	0.0039891	1	0.001761	1	0.002437	1	0.021147
SM d18:0/24:0	SM	0.1087	0.0133216	1	0.002492	1	0.003025	1	0.017893
PC36:4p(20:4)	PC	1	0.0022282	0.5567396	0.011348	1	0.011235	1	0.002536
PC36:4p(20:3)	PC	1	0.0014756	1	-0.000805	1	0.001175	1	0.008047
PC36:3p(20:3)	PC	1	-8.71E-07	1	0.005886	1	0.006733	1	-0.00041
PC38:3p(20:3)	PC	0.264841	0.0150974	1	-0.001161	1	6.99E-05	1	0.003093
PC38:4p(20:4)	PC	0.329907	0.0078245	1	-0.001123	1	0.000199	1	0.007155
PC34:2(16:1)	PC	1	-3.16E-06	1	0.002798	1	0.00408	1	0.00159
PC34:2(16:0)	PC	1	0.0023221	1	0.003049	1	0.001961	0.000141	0.033458
PC34:1(16:1)	PC	1	0.0007037	1	-0.000553	1	3.20E-05	0.000134	0.036573
PC34:1(16:0)	PC	1	-7.42E-07	0.031521	0.018578	0.109763	0.016731	1	0.009533
PC36:5(20:5)	PC	1	0.0081314	1	-0.000462	1	0.001144	1	0.0037
PC36:5(20:4)	PC	1	2.58E-06	1	-0.001187	1	0.000607	1	0.005143
PC36:4(20:4)	PC	1	5.99E-05	1	0.003653	1	0.004048	1	0.002703
PC36:4(20:3)	PC	0.169282	0.0081941	0.0153049	0.019979	0.017371	0.02144	1	0.010165
PC36:3(20:3)	PC	0.108238	0.0067623	1	-0.000911	1	0.000724	1	0.009772
PC38:6(22:6)	PC	1	0.0032519	1	0.001072	1	0.002912	1	0.002142
PC38:6(22:5)	PC	1	5.38E-05	0.0010294	0.026772	0.000944	0.029063	1	-0.001268
PC38:6(20:4)	PC	1	3.62E-07	1	0.00083	1	0.003974	0.019919	0.019802
PC38:6(20:3)	PC	1	7.14E-06	0.9330735	0.010059	0.603419	0.012466	1	-5.53E-06

PC38:5(22:5)	PC	1	0.0075885	0.3951475	0.012201	0.590676	0.012535	1	0.000616
PC38:5(22:4)	PC	1	0.0006199	1	-0.001227	1	7.97E-05	1	-0.001194
PC38:5(20:4)	PC	1	0.0018147	1	0.005137	1	0.007432	1	0.000746
PC38:5(20:3)	PC	1	-8.65E-06	0.0141547	0.020687	0.005747	0.024368	1	0.002026
PC38:4(20:4)	PC	1	4.73E-06	1	0.002429	1	0.004464	1	0.000623
PC38:4(20:3)	PC	1	1.35E-06	0.0003861	0.030051	0.001466	0.027868	1	0.007328
PC38:3(20:3)	PC	0.001947	0.0112045	0.0006767	0.02776	0.003133	0.025882	0.668819	0.017311
PC40:7(22:6)	PC	1	7.85E-06	0.0190998	0.019856	0.030329	0.020008	1	0.000586
PC40:7(22:5)	PC	1	0.0002185	0.5710734	0.011265	0.302144	0.014185	1	0.001489
PC40:7(20:4)	PC	1	-2.13E-06	1	-0.001029	1	0.000493	1	0.000308
PC40:7(20:3)	PC	1	7.03E-08	1	0.004092	1	0.005759	1	-0.001116
PC40:6(22:6)	PC	0.077377	0.0053797	0.5578644	0.01088	1	0.010948	1	0.012067
PC40:6(22:5)	PC	1	9.92E-05	0.0012717	0.02702	0.002555	0.026414	1	0.00178
PC40:6(20:4)	PC	1	2.65E-05	1	0.000924	1	0.002537	1	-0.000827
PC40:6(20:3)	PC	1	0.0004724	1	0.008327	1	0.009072	1	-0.00029
PC40:5(22:5)	PC	0.012787	0.0099789	0.0001737	0.032208	0.000923	0.029122	0.689048	0.01085
PC40:5(20:4)	PC	1	0.0097932	1	0.000983	1	0.00239	1	-0.001311
PC40:5(20:3)	PC	1	-1.21E-05	0.7328367	0.010669	1	0.01023	1	0.004607
PC40:4(20:4)	PC	0.567069	0.0136422	1	0.002632	1	0.004141	1	0.00053
PC40:4(20:3)	PC	1	0.0113567	1	0.006805	1	0.006211	1	0.005524
PC40:3(20:3)	PC	1	0.0073248	1	0.005033	1	0.005124	1	0.003678
14:0-carnitine	Carnitine	1	8.85E-07	1	-0.000731	1	0.00106	1	0.004394
16:0-carnitine	Carnitine	1	3.07E-05	1	-0.001256	1	0.000139	1	-0.000925
18:0-carnitine	Carniti	1	-4.80E-07	1	0.001803	1	0.002894	1	-0.000985

	ne								
18:1-carnitine	Carnitine	1	0.0007665	1	0.000959	1	0.001842	1	0.000766
18:2-carnitine	Carnitine	1	1.42E-05	0.524052	0.011651	0.690665	0.012314	1	-0.000233
LPI16:1	LPI	1	0.0029372	1	0.008228	1	0.011281	1	0.003018
LPI16:0	LPI	0.027902	0.0077426	1	-0.0012	1	1.52E-06	0.612203	0.011245
LPI18:0	LPI	1	0.0084858	1	-0.000215	1	0.000785	1	0.000979
LPI20:4	LPI	0.053048	0.0168268	1	0.00871	1	0.009621	1	-0.000754
LysoPC18:1	LysoPC	1	0.0010584	0.1299089	0.014665	0.120931	0.016553	1	0.004342
LysoPC18:0	LysoPC	0.025183	0.02664	1	0.001019	1	0.003526	1	0.007317
LysoPC20:5	LysoPC	1	0.0032176	1	-0.001341	1	1.68E-05	1	0.00141
LysoPC20:4	LysoPC	1	3.10E-06	0.0137927	0.020788	0.029867	0.020129	1	0.002679
LysoPC20:3	LysoPC	1	0.0025895	1	0.006356	1	0.009005	1	0.001805
LysoPC22:6	LysoPC	1	3.07E-06	1	0.007947	1	0.008327	1	0.001577
LysoPC22:5	LysoPC	1	2.25E-05	1	0.001875	1	0.003249	1	-0.001344
DAG32:2(18:2/14:0)	DAG	1	3.26E-05	1	-0.001285	1	1.76E-06	1	0.00135
DAG34:0(16:0/18:0)	DAG	1	0.0016747	1	-0.001256	1	4.25E-05	1	-0.000808
DAG34:2(16:0/18:2)	DAG	1	0.0016467	1	-0.000784	1	0.000252	1	0.003278
DAG36:1(18:1/18:0)	DAG	1	4.09E-06	1	-0.001276	1	7.54E-05	1	-0.001353
DAG36:2(18:2/18:0)	DAG	1	2.72E-06	1	-0.001238	1	0.000307	1	0.001116

DAG36:3(18:2/18:1)	DAG	1	0.0011054	1	-0.000528	1	0.0013	1	0.001848
DAG38:6(18:1/20:5)	DAG	1	0.0027427	1	0.002673	1	0.004851	1	0.00093
DAG38:5(18:0/20:5)	DAG	1	-1.93E-06	1	0.002646	1	0.005218	1	0.01441
DAG38:4(18:0/20:4)	DAG	1	-6.09E-06	1	0.001983	1	0.004984	0.654386	0.011
DAG38:4(18:1/20:3)	DAG	1	0.0001845	1	-0.000824	1	0.00068	1	-0.000787
DAG40:6(18:1/22:5)	DAG	1	0.0007426	1	-7.55E-05	1	0.0021	1	0.002255
DAG40:6(18:0/22:6)	DAG	1	0.0007567	1	-0.001274	1	0.000337	1	0.002692
DAG40:5(18:0/22:5)	DAG	1	0.0051841	1	-0.001201	1	7.46E-05	1	0.000257
CE-14:1	CE	1	0.0039067	1	0.004858	1	0.006559	1	0.003353
CE-14:0	CE	0.002077	0.0066929	1	0.008019	1	0.007272	0.184026	0.019839
CE-15:1	CE	1	0.0023538	0.0857173	0.015703	0.392539	0.013532	0.001192	0.036798
CE-15:0	CE	1	0.0022146	0.0034726	0.023702	0.058189	0.018392	0	0.050826
CE-16:2	CE	0.000112	0.0159817	0.0186423	0.019729	0.049667	0.018822	0.010402	0.032331
CE-16:1	CE	1	0.0073266	1	-0.000917	1	1.43E-06	0.000175	0.044413
CE-16:0	CE	1	0.0001626	0.2014167	0.01352	0.468553	0.013092	0.133201	0.025107
CE-17:1	CE	1	0.0003535	0.3533849	0.012115	0.862819	0.011585	0.029898	0.030244
CE-17:0	CE	1	0.0034671	1	0.006601	1	0.006014	0.000997	0.041732
CE-18:3	CE	0.001521	0.0148296	3.46E-08	0.055745	1.06E-07	0.054296	1	0.016875

CE-18:2	CE	0.014374	0.0127298	0.0003863	0.029206	0.00024	0.032639	1	0.016461
CE-18:1	CE	0.006773	0.0101147	0.0028877	0.024014	0.003418	0.025655	0.756168	0.022109
CE-18:0	CE	0.425765	0.0058097	0.3001522	0.012318	0.657632	0.012253	0.194123	0.025234
CE-19:1	CE	0.014578	0.0084223	1	0.005994	1	0.006569	0.637026	0.022211
CE-19:0	CE	0.080672	0.0052888	1	0.008144	1	0.008624	1	0.015613
CE-20:5	CE	0.138593	0.0090787	0.0097249	0.021595	0.029364	0.020337	0.240626	0.021394
CE-20:4	CE	0.184195	0.0079988	0.104087	0.015129	0.104044	0.016867	1	0.01502
CE-20:3	CE	0.012536	0.0062193	0.0946046	0.015447	0.14524	0.016045	0.398713	0.0224
CE-20:2	CE	0.003165	0.0106246	0.0054034	0.022742	0.023547	0.020685	0.003503	0.036788
CE-20:1	CE	0.288169	0.0097072	1	0.005278	1	0.006559	1	0.014116
CE-20:0	CE	1	0.0026268	1	0.009495	1	0.010586	1	0.014269
CE-21:1	CE	1	0.014486	0.0019366	0.025442	0.002916	0.026175	1	0.00877
CE-21:0	CE	1	0.0094712	0.1587139	0.014242	0.223981	0.014954	1	0.006272
CE-22:6	CE	0.001034	0.0080063	0.0002575	0.030811	0.000372	0.031566	0.130007	0.025814
CE-22:5	CE	0.00051	0.0083651	0.0001145	0.032908	0.000274	0.03233	0.050813	0.028719
CE-22:4	CE	0.136767	0.0038954	0.0019662	0.024689	0.007607	0.023606	1	0.016218
CE-22:3	CE	0.458421	0.0057238	1	0.003196	1	0.003911	1	0.008908
CE-22:2	CE	0.21441	0.0064383	0.3004929	0.012672	0.338383	0.013996	1	0.012406
CE-22:1	CE	1	1.28E-05	1	0.003226	1	0.004346	1	0.005754
CE-22:0	CE	1	0.0090173	1	0.001049	1	0.002574	1	-0.001234
TAG44:1(14:0)	TAG	1	-2.14E-05	1	0.000359	1	0.002138	1	0.000262
TAG44:1(16:0)	TAG	1	8.38E-05	1	-8.51E-05	1	0.001817	1	0.001237
TAG44:1(16:1)	TAG	1	-8.31E-06	1	0.003962	1	0.006438	1	0.001996
TAG46:2(18:2)	TAG	1	0.0010266	1	-0.001331	1	1.83E-05	1	-0.001062
TAG46:1(14:1)	TAG	1	-8.85E-06	1	0.004469	1	0.006705	1	2.46E-05
TAG48:4(16:1)	TAG	1	0.0071762	1	-0.001344	1	4.89E-05	1	0.001893

TAG48:3(16:1)	TAG	0.091304	0.0094903	1	-0.001239	1	0.000434	1	0.006475
TAG48:3(18:2)	TAG	1	0.0066032	1	-0.000626	1	0.000499	1	0.000266
TAG48:2(16:0)	TAG	0.121333	0.0106152	1	-0.001085	1	0.000107	1	0.006688
TAG48:2(16:1)	TAG	1	0.0031083	1	-0.001352	1	0.000441	0.043414	0.018273
TAG48:2(18:1)	TAG	0.265439	0.0027426	1	0.001405	1	0.001525	0.657513	0.011184
TAG48:2(18:2)	TAG	0.074225	0.0155852	1	-0.001276	1	8.13E-05	1	0.000377
TAG48:1(16:0)	TAG	1	0.0029838	1	-0.00129	1	0.00036	1	0.005262
TAG48:1(16:1)	TAG	0.146346	0.005296	1	0.004551	1	0.008086	1	0.010096
TAG48:1(18:0)	TAG	1	0.0042513	1	-0.001271	1	0.000324	1	0.001758
TAG50:4(16:2)	TAG	0.025427	0.0209168	1	0.003454	1	0.003837	1	8.05E-05
TAG50:4(18:2)	TAG	0.066297	0.0191835	1	-0.001195	1	5.96E-06	1	0.006588
TAG50:3(16:0)	TAG	0.01515	0.0155305	1	-0.000841	1	0.00085	1	0.004084
TAG50:3(16:1)	TAG	0.074493	0.0104811	1	-0.00094	1	0.000814	1	0.014079
TAG50:3(16:2)	TAG	0.137843	0.0164727	1	0.000797	1	0.001759	1	0.005453
TAG50:3(18:1)	TAG	0.010174	0.0251368	1	-0.001341	1	0.0001	1	0.009606
TAG50:3(18:2)	TAG	0.01586	0.0232067	1	-0.00078	1	0.000433	1	0.008662
TAG50:2(16:0)	TAG	1	0.0078319	1	-0.000196	1	0.00124	1	0.002147
TAG50:2(16:1)	TAG	0.120924	0.0054791	1	-0.001032	1	0.000602	1	0.014641
TAG50:2(16:2)	TAG	1	0.0001443	1	-0.000221	1	0.000792	1	0.002655
TAG50:2(18:1)	TAG	1	0.0006581	1	-0.00126	1	5.52E-06	1	0.012471
TAG50:2(18:2)	TAG	1	0.0070628	1	-0.001297	1	5.35E-05	1	0.000385
TAG50:1(16:0)	TAG	1	0.0070422	1	0.002877	1	0.004415	1	0.002535
TAG50:1(16:1)	TAG	1	0.0064056	1	0.002007	1	0.003993	1	0.01065
TAG50:1(18:0)	TAG	1	0.0061718	1	-0.000477	1	0.001106	1	0.005515
TAG50:0(18:0)	TAG	1	0.0053495	1	0.002517	1	0.004028	1	0.003411
TAG51:3(17:1)	TAG	0.008268	0.0132595	1	-0.001202	1	0.00027	1	0.014331

TAG51:2(15:0)	TAG	0.022024	0.0004988	1	0.000127	1	0.000787	0.962294	0.021311
TAG51:2(17:0)	TAG	0.02367	0.0128337	1	-0.001277	1	0.000276	1	0.008319
TAG51:0(17:0)	TAG	0.029927	0.0098011	1	0.00041	1	0.002622	1	0.010365
TAG52:6(16:1)	TAG	0.00275	0.0266446	1	0.002343	1	0.004225	1	0.002461
TAG52:6(16:2)	TAG	0.042975	0.019093	1	0.001402	1	0.001338	1	0.00971
TAG52:6(16:3)	TAG	0.004621	0.0236563	1	-0.001261	1	0.000252	1	0.000205
TAG52:6(18:3)	TAG	0.002397	0.0266083	1	0.004094	1	0.00659	1	0.001816
TAG52:5(16:0)	TAG	0.003306	0.0241921	1	-0.001142	1	0.000794	1	0.007618
TAG52:5(16:1)	TAG	0.04431	0.0185822	1	-0.001307	1	2.27E-05	1	0.012056
TAG52:5(16:2)	TAG	0.18726	0.0162715	1	0.001268	1	0.001174	1	0.011201
TAG52:5(16:3)	TAG	0.177735	0.0146936	1	-0.001342	1	0.000101	1	0.00323
TAG52:5(18:2)	TAG	0.01237	0.0225095	1	-0.001255	1	2.93E-05	1	0.011763
TAG52:5(18:3)	TAG	0.00389	0.0231932	1	-0.00062	1	0.001537	1	0.006384
TAG52:4(16:0)	TAG	0.197964	0.0157684	1	-0.001001	1	7.00E-07	1	0.016163
TAG52:4(16:1)	TAG	0.232695	0.0198589	1	-0.000937	1	0.000179	1	0.01091
TAG52:4(16:2)	TAG	0.063553	0.0166259	1	0.001729	1	0.001746	1	0.016012
TAG52:4(18:1)	TAG	0.128449	0.0179838	1	-0.000353	1	0.000373	1	0.012131
TAG52:4(18:2)	TAG	0.111845	0.0172886	1	0.000341	1	0.000512	1	0.019049
TAG52:4(18:3)	TAG	0.055317	0.0185884	1	-0.001085	1	0.000846	1	0.008115
TAG52:3(16:0)	TAG	0.316805	0.0162219	1	-0.000914	1	0.000114	1	0.009587
TAG52:3(16:1)	TAG	1	-0.009018	1	-0.001323	1	5.59E-05	1	0.012755
TAG52:3(16:2)	TAG	0.051492	0.0198302	1	0.003483	1	0.002991	1	0.008
TAG52:3(18:1)	TAG	0.419572	0.0167431	1	-0.001326	1	8.22E-07	1	0.008501
TAG52:3(18:2)	TAG	0.279965	0.0171338	1	-0.001353	1	3.45E-05	1	0.009404
TAG52:1(16:1)	TAG	0.755352	0.0112104	1	-0.001266	1	1.15E-05	1	0.002509
TAG53:5(17:1)	TAG	0.129155	0.0146361	1	-0.000662	1	0.00178	1	0.020077

TAG53:5(18:3)	TAG	0.075928	0.0163886	1	0.002599	1	0.00554	1	0.007609
TAG53:3(16:0)	TAG	1	0.0105204	1	0.004903	1	0.00484	1	0.008889
TAG53:3(19:1)	TAG	1	0.0118897	1	0.004313	1	0.005142	1	0.006254
TAG53:2(17:1)	TAG	1	0.0081553	1	0.000895	1	0.002137	1	0.005927
TAG53:2(19:0)	TAG	0.741344	0.012624	1	0.000222	1	0.000881	1	0.001645
TAG53:2(19:1)	TAG	1	0.0052136	1	0.002413	1	0.00351	1	0.006406
TAG54:8(22:6)	TAG	0.255328	0.0146564	1	-0.000512	1	0.00037	1	0.003957
TAG54:7(20:4)	TAG	0.00577	0.0234397	1	-0.001261	1	0.000204	1	0.003955
TAG54:7(20:5)	TAG	0.00403	0.0234459	1	-0.001299	1	4.57E-05	1	0.006436
TAG54:7(22:6)	TAG	0.571896	0.0108414	1	1.88E-06	1	0.001341	1	0.004463
TAG54:6(18:2)	TAG	0.082386	0.0177142	1	-0.000448	1	0.002523	1	0.017154
TAG54:6(18:3)	TAG	0.435367	0.0118257	1	0.002649	1	0.006604	1	0.011846
TAG54:6(20:4)	TAG	0.433999	0.0156502	1	-0.00103	1	0.000127	1	0.00493
TAG54:5(16:0)	TAG	1	0.0080918	1	-0.000131	1	0.000769	1	0.008698
TAG54:5(16:1)	TAG	1	0.0075128	1	0.00075	1	0.001901	1	0.004319
TAG54:5(16:2)	TAG	0.503469	0.0134999	1	0.002259	1	0.001782	1	0.014477
TAG54:5(18:1)	TAG	1	0.0099872	1	-0.001356	1	0.00058	1	0.014656
TAG54:5(18:2)	TAG	1	0.0087181	1	-0.001266	1	0.001047	1	0.018149
TAG54:5(18:3)	TAG	0.504585	0.0130739	1	-0.000466	1	0.002245	1	0.0105
TAG54:5(20:4)	TAG	1	0.0055861	1	0.000497	1	0.001441	1	0.006476
TAG54:4(16:0)	TAG	1	0.0117709	1	0.003125	1	0.003501	1	0.005294
TAG54:4(16:1)	TAG	1	1.37E-05	1	-0.000451	1	0.000598	1	0.006418
TAG54:4(16:2)	TAG	1	1.22E-05	1	-0.001318	1	0.000369	1	0.006328
TAG54:4(18:0)	TAG	1	0.0108629	1	-0.000956	1	0.001053	1	0.007875
TAG54:4(18:1)	TAG	1	0.0037836	1	-0.001354	1	0.000424	1	0.015363
TAG54:4(18:2)	TAG	1	0.0067657	1	-0.001148	1	0.000909	1	0.010747

TAG54:4(18:3)	TAG	1	0.0086799	1	0.000342	1	0.003089	1	0.010439
TAG54:3(16:0)	TAG	1	5.03E-06	1	0.000533	1	0.002041	1	-0.001277
TAG54:3(18:1)	TAG	1	-1.39E-05	1	-0.001383	1	2.27E-05	1	0.000158
TAG54:3(18:2)	TAG	1	0.0058877	1	-0.00131	1	8.86E-06	1	0.007437
TAG54:2(18:0)	TAG	1	-1.19E-05	1	-0.001291	1	0.000193	1	-0.00061
TAG54:2(18:2)	TAG	1	0.0016175	1	-0.001352	1	1.44E-05	1	0.004435
TAG55:6(19:3)	TAG	1	0.0065082	1	-0.001104	1	0.000629	1	0.003173
TAG55:5(18:2)	TAG	0.177428	0.0159932	1	-0.001361	1	0.000329	1	0.01302
TAG56:8(20:6)	TAG	1	0.006912	1	-0.001337	1	4.63E-07	1	0.009682
TAG56:8(18:2)	TAG	0.084646	0.0169843	1	-0.001124	1	1.24E-09	1	0.013066
TAG56:7(18:3)	TAG	0.045618	0.0184029	1	-0.000823	1	0.001391	1	0.008702
TAG56:7(22:5)	TAG	1	0.0058665	1	-0.001376	1	0.00019	1	0.013249
TAG56:6(18:2)	TAG	0.370701	0.0147499	1	-0.001231	1	0.000761	1	0.013928
TAG56:6(22:4)	TAG	0.32512	0.0154655	1	-0.000653	1	0.000449	1	0.00448
TAG56:6(22:5)	TAG	1	0.0072184	1	-0.001355	1	0.000115	1	0.011438
TAG56:5(18:1)	TAG	1	0.0069534	1	-0.001357	1	0.000254	1	0.006699
TAG56:5(18:2)	TAG	0.665206	0.0142487	1	-0.001382	1	0.000312	1	0.015192
TAG56:5(20:2)	TAG	1	0.0107935	1	-0.001126	1	0.000996	1	0.016313
TAG56:5(22:4)	TAG	1	0.008218	1	-0.000364	1	0.000835	1	0.005118
TAG56:4(20:0)	TAG	1	0.0060024	1	-0.001371	1	8.09E-05	0.973765	0.010561
TAG56:4(20:2)	TAG	1	-1.31E-05	1	-0.001362	1	7.35E-07	1	0.005749
TAG58:8(22:5)	TAG	1	0.0070971	1	-0.00046	1	0.002251	1	0.006361
TAG58:7(20:4)	TAG	0.533369	0.0130782	1	-0.001347	1	3.19E-05	1	0.005474
TAG58:7(22:5)	TAG	1	0.0058942	1	-0.000663	1	0.001554	1	0.008064

Age-dependent sex differences for each lipid-omics trait are provided based on GAM age-by-sex interaction P value significance and effect size estimated using Cohen's f2. Non-linear sex differences over the whole age span were estimated using GAMs, where the interaction term was omitted; the sex term significance and effect size

(Cohen's f2) are provided. The non-linear age effect for both sexes combined was estimated using GAMs, where the interaction term was omitted; the age term significance and effect size (Cohen's f2) are provided.

Supplementary Table S6 The proportion of lipid variance is explained by age, sex, and their interaction and K-means clustering for distinguished patterns.

Lipids	Null_model	Sex	Age	Sex:age	Rest	Class	Cluster
Cer d18:1/16:0	0.191115824	0.0385062	0.0608931	0.10943314	0.60005175	Cer	3
Cer d18:0/16:0	0.091419132	0.0339326	0.0016603	0.05013478	0.82285314	Cer	5
Cer d18:1/18:0	0.185134025	0.157595	0.0893658	0.0377991	0.53010609	Cer	1
Cer d18:0/18:0	0.153723622	0.0302592	0.0077574	0.0369898	0.77126999	Cer	5
Cer d18:1/22:0	0.16655976	0.0270715	0.0853386	0.19172053	0.52930965	Cer	3
Cer d18:0/22:0	0.325980435	0.0699227	0.1301716	0.19900612	0.27491908	Cer	3
Cer d18:1/24:1	0.072625009	0.0714773	0.0492767	0.00444046	0.80218057	Cer	5
Cer d18:1/24:0	0.092087478	0.0057006	0.073605	0.30525635	0.52335057	Cer	3
FFA22:6	0.24314325	0.0144409	0.0316282	0	0.71078762	FFA	5
FFA22:5	0.072883705	0.0781529	0.0063935	0.00042146	0.84214843	FFA	5
FFA22:4	0.049819621	0.0186956	0.0036817	0.00154035	0.9262628	FFA	5
FFA18:2	0.134593346	0.0574144	0.0095015	0.0206227	0.77786809	FFA	5
FFA18:1	0.059657689	0.1074242	0.0253641	0.00013045	0.8074235	FFA	5
FFA18:0	0.242770522	0.0826568	0.0782367	0.01429646	0.58203948	FFA	5
FFA16:1	0.032062363	0.0035913	0.0356538	0.03038966	0.89830289	FFA	5
FFA16:0	0.041432842	0.0186076	0.0838266	0.03093767	0.82519537	FFA	5
PG38:6(22:5)	0.022598591	0.0391746	0.027623	0.03475032	0.87585345	PG	5
PG38:5(22:5)	0.036012869	0.0004094	0.0137963	0.05147065	0.89831077	PG	5
PG36:3(18:1)	0.067313759	0.0342792	0.1088954	0.02352206	0.76598955	PG	5
PG36:2	0.0586509	0.0187823	0.1801375	0.05370614	0.6887232	PG	3
PG36:1	0.095224159	6.80E-06	0.0928866	0.05339657	0.7584859	PG	3
PG38:6	0.181179457	0.0034216	0.0167816	0.00726909	0.79134829	PG	5
PG38:5	0.262063104	0.0007347	0.0525421	0.00194371	0.68271639	PG	5
PG38:4	0.19501391	0.0204184	0.0098587	0.02284892	0.75186014	PG	5
PG38:3	0.206985333	0.0064459	0.0186199	0	0.76794893	PG	5
PE32:1	0.043686585	0.0113417	6.93E-05	0.00065907	0.94424329	PE	5
PE34:2	0.084417731	0.0276214	0.006049	0.04794149	0.83397034	PE	5
PE36:3	0.044208157	0.0961869	0.0498327	0.02746492	0.78230728	PE	5
PE36:2	0.043023787	0.1151003	0.0862463	0.05928655	0.69634311	PE	5
PE42:3p	0.125967049	0.0097985	0.1773892	0.01526557	0.67157967	PE	3
PE40:5p	0.45401061	0.0785733	0.0601906	0.02457188	0.38265361	PE	5
PE38:5p	0.07766506	0.005896	0.2106779	0.00761791	0.6981431	PE	2
PE38:4p	0.081957694	0.0024056	0.1384954	0.00303802	0.77410331	PE	3
PE36:1p	0.539794302	0.0533023	0.0248987	0.0204008	0.36160386	PE	5
PE38:5p(20:4)	0.108947115	0.0016387	0.158721	0.00169788	0.72899526	PE	3
PE38:4p(20:4)	0.082563725	0.0007644	0.0961651	0.00341023	0.81709657	PE	5
PE40:5p(22:5)	0.456532588	0.0604802	0.0339488	0.03754936	0.41148907	PE	5
PC32:2	0.071350076	0.5766489	0.0161596	0.02078813	0.31505339	PC	4
PC32:1	0.073553799	0.0092414	0.2877214	0.00648296	0.62300045	PC	2
PC32:0	0.12135282	0.039692	0.2159465	0	0.62300864	PC	2
PC34:3	0.047648128	0.4538738	0.030697	0.08867177	0.37910931	PC	4

PC36:6	0.175869144	0.4846261	0.1104682	0.15937805	0.06965849	PC	4
PC36:5	0.07212588	0.1130852	0.1159209	0.1326945	0.56617353	PC	3
PC38:5	0.101633664	0.2706006	0.0209676	0.0940267	0.51277144	PC	1
PC40:7	0.235627885	0.350436	0.0144284	0.02473757	0.37477012	PC	1
PC40:6	0.17295001	0.2403376	0.0322097	0.04661919	0.50788357	PC	1
PC40:5	0.10917999	0.3354902	0.009683	0.05282011	0.49282676	PC	1
PC40:4	0.099348951	0.0762268	0.0004115	0.01323026	0.81078245	PC	5
PC40:3	0.013675351	0.0206342	0.010789	0.01649193	0.93840954	PC	5
SM d18:0/18:0	0.489552334	0.1314845	0.3493812	0.00815978	0.02142225	SM	2
SM d18:1/18:0	0.148897412	0.1873659	0.4452006	0.01373511	0.20480092	SM	2
SM d18:1/20:0	0.156006226	0.0976678	0.3336349	0.00073592	0.41195516	SM	2
SM d18:1/21:0	0.086894418	0.4707362	0.3362556	0.09638007	0.00973378	SM	4
SM d18:1/22:0	0.16389857	0.0211238	0.2397141	0.05555558	0.51970801	SM	2
SM d18:1/23:0	0.125613247	0.1678757	0.2220507	0.14725247	0.33720782	SM	2
SM d18:1/24:0	0.118335457	0.0189281	0.2731652	0.05925126	0.53031996	SM	2
SM d18:0/24:0	0.144300264	0.0270463	0.2378045	0.11994295	0.47090597	SM	2
PC36:4p(20:4)	0.030027036	0.107958	0.0181713	0.00736105	0.8364827	PC	5
PC36:4p(20:3)	0.068189377	0.0178781	0.0519386	0.01197287	0.85002112	PC	5
PC36:3p(20:3)	0.037356558	0.0807039	0.0003218	0.00453908	0.87707867	PC	5
PC38:3p(20:3)	0.210688705	0.0006679	0.0827751	0.1009892	0.6048791	PC	3
PC38:4p(20:4)	0.185225533	0.0089562	0.0922896	0.06704771	0.64648096	PC	3
PC34:2(16:1)	0.228890871	0.0445923	0.0380757	0.00783498	0.68060619	PC	5
PC34:2(16:0)	0.055014906	0.0196848	0.3450012	0.03781806	0.54248104	PC	2
PC34:1(16:1)	0.187129656	0.0014148	0.2510361	0.01046749	0.54995196	PC	2
PC34:1(16:0)	0.050751817	0.1701828	0.1175426	0.01823244	0.64329043	PC	1
PC36:5(20:5)	0.076270534	0.0335604	0.1785634	0.07349715	0.63810856	PC	3
PC36:5(20:4)	0.042910801	0.0115277	0.0506119	0.01311056	0.88183907	PC	5
PC36:4(20:4)	0.050379783	0.0459493	0.0285571	0.02964258	0.84547118	PC	5
PC36:4(20:3)	0.187070176	0.2394973	0.1353477	0.07520175	0.3628831	PC	1
PC36:3(20:3)	0.59048471	0.0026899	0.1145983	0.04827022	0.24395687	PC	3
PC38:6(22:6)	0.072841982	0.0388485	0.0527767	0.02901746	0.80651542	PC	5
PC38:6(22:5)	0.26758952	0.2813171	0.0143721	0.02936031	0.40736092	PC	1
PC38:6(20:4)	0.14296341	0.0266417	0.1433806	0.01506986	0.67194444	PC	3
PC38:6(20:3)	0.029068455	0.1240299	0.0075123	0.02628577	0.81310361	PC	5
PC38:5(22:5)	0.201476958	0.1318674	0.0298786	0.11877028	0.51800676	PC	5
PC38:5(22:4)	0.254734926	1.80E-05	0.0113029	0.03619556	0.69774868	PC	5
PC38:5(20:4)	0.304551526	0.0516932	0.0047684	0.00042348	0.63856339	PC	5
PC38:5(20:3)	0.132011425	0.2465364	0.0069457	0.01150718	0.60299928	PC	1
PC38:4(20:4)	0.045990659	0.0399139	0.0093205	0.03634356	0.86843141	PC	5
PC38:4(20:3)	0.044209199	0.2618904	0.1191369	0.00031927	0.57444424	PC	1
PC38:3(20:3)	0.418904244	0.2850066	0.2248782	0.07121093	0	PC	4
PC40:7(22:6)	0.168048996	0.1882995	0.0177914	0.00411342	0.62174675	PC	1
PC40:7(22:5)	0.440271032	0.1156172	0.0084813	0.04453015	0.39110026	PC	5
PC40:7(20:4)	0.080384382	0.0010223	0.0032642	0.00013661	0.91519248	PC	5

PC40:7(20:3)	0.061661779	0.0482426	3.96E-06	0.02601827	0.8640734	PC	5
PC40:6(22:6)	0.156543002	0.1361368	0.1129414	0.05367223	0.54070664	PC	1
PC40:6(22:5)	0.720567667	0.2206782	0.0515049	0.00163794	0.00561123	PC	1
PC40:6(20:4)	0.171576427	0.0137918	9.84E-05	0.00053939	0.81399396	PC	5
PC40:6(20:3)	0.059199126	0.080605	0.0295409	0.00963015	0.8210248	PC	5
PC40:5(22:5)	0.065523258	0.3031768	0.1357653	0.11067611	0.38485856	PC	4
PC40:5(20:4)	0.196355969	0.0343192	0.0002602	0.06881409	0.70025061	PC	5
PC40:5(20:3)	0.083412	0.0997181	0.0954102	9.43E-07	0.72145871	PC	5
PC40:4(20:4)	0.251283128	0.0295198	0.0017185	0.05607472	0.66140384	PC	5
PC40:4(20:3)	0.028185622	0.0709269	0.0495618	0.08010997	0.77121566	PC	5
PC40:3(20:3)	0.032968648	0.0734949	0.0434516	0.07655939	0.77352544	PC	5
14:0-carnitine	0.002351684	0.0014499	0.0386222	0.00826234	0.94931386	Carnitine	5
16:0-carnitine	0.074325567	0.0032177	0.0079912	0.01883883	0.89562677	Carnitine	5
18:0-carnitine	0.009844229	0.0215919	0.0195348	0.00850131	0.94052774	Carnitine	5
18:1-carnitine	0.014877593	0.0149837	0.0154072	0.00367938	0.95105218	Carnitine	5
18:2-carnitine	0.071959966	0.101545	0.079422	0.05826306	0.68881001	Carnitine	5
LPI16:1	0.188693383	0.0502352	0.0001801	0.00552675	0.75536459	LPI	5
LPI16:0	0.232410363	0.0042371	0.0371025	0.05758403	0.668666	LPI	5
LPI18:0	0.167203631	0.0006139	0.000608	0.13602665	0.69554782	LPI	5
LPI20:4	0.023896802	0.12059	0.0448434	0.18266158	0.62800819	LPI	3
LysoPC18:1	0.28828398	0.2477841	0.0789208	0.00640652	0.37860464	LysoPC	1
LysoPC18:0	0.25660835	0.043738	0.1406199	0.23693522	0.3220985	LysoPC	3
LysoPC20:5	0.13525339	0.0174122	0.0827275	0.05764862	0.7069583	LysoPC	5
LysoPC20:4	0.024712896	0.2746194	0.1113209	0.01609407	0.57325268	LysoPC	1
LysoPC20:3	0.121749917	0.1049606	0.0021036	0.01654389	0.75464194	LysoPC	5
LysoPC22:6	0.21660649	0.0990495	0.0928732	0.03016127	0.56130953	LysoPC	5
LysoPC22:5	0.093228018	0.0404382	0.0156615	0.03155466	0.81911766	LysoPC	5
DAG32:2(18:2/14:0)	0.077638131	0.0075651	0.0367602	0.00541897	0.87261756	DAG	5
DAG34:0(16:0/18:0)	0.192504008	3.27E-05	8.96E-05	0.02324573	0.78412799	DAG	5
DAG34:2(16:0/	0.103881671	0.0089513	0.0349375	0.00610902	0.84612045	DAG	5

18:2)							
DAG36:1(18:1/18:0)	0.112558621	0.0131826	0.0001459	0.00932238	0.86479052	DAG	5
DAG36:2(18:2/18:0)	0.140570984	0.0143119	0.0427601	0.0078055	0.79455148	DAG	5
DAG36:3(18:2/18:1)	0.084691974	8.69E-05	0.0929569	0.00040309	0.82186115	DAG	5
DAG38:6(18:1/20:5)	0.074445596	7.35E-06	0.0540916	0.00894285	0.86251261	DAG	5
DAG38:5(18:0/20:5)	0.100273746	0.0373039	0.1008594	0.01081587	0.75074703	DAG	5
DAG38:4(18:0/20:4)	0.103279804	0.0229884	0.097031	0.00238162	0.77431923	DAG	5
DAG38:4(18:1/20:3)	0.085524573	0.0001297	0.0119783	0.02718017	0.87518729	DAG	5
DAG40:6(18:1/22:5)	0.127046708	0.0001268	0.0115148	0.04265866	0.81865298	DAG	5
DAG40:6(18:0/22:6)	0.013135117	0.0096784	0.001144	0.01526284	0.9607797	DAG	5
DAG40:5(18:0/22:5)	0.029843118	0.002449	0.0183231	0.03163784	0.91774701	DAG	5
CE-14:1	0.049259497	0.044899	0.0962834	0.09073075	0.71882728	CE	3
CE-14:0	0.108888814	0.0738352	0.2191013	0.0550352	0.5431395	CE	2
CE-15:1	0.083472618	0.1440749	0.4450556	0.03612041	0.29127648	CE	2
CE-15:0	0.00156735	0.1766568	0.5497778	0.03543621	0.23656189	CE	2
CE-16:2	0.108199939	0.1933671	0.325375	0.13813681	0.23492121	CE	2
CE-16:1	0.251131798	0.0028724	0.4336075	0.04438989	0.26799841	CE	2
CE-16:0	0.030879295	0.1330699	0.3283991	0.02449698	0.48315471	CE	2
CE-17:1	0.01410495	0.1175141	0.3809194	0.02594966	0.46151195	CE	2
CE-17:0	0.023329718	0.0360117	0.3960598	0.03062721	0.51397158	CE	2
CE-18:3	0.059181892	0.4389857	0.1964883	0.11414964	0.19119445	CE	4
CE-18:2	0.16089329	0.2902478	0.2768484	0.09740756	0.17460296	CE	4
CE-18:1	0.114976654	0.2266088	0.3491771	0.08860651	0.22063097	CE	2
CE-18:0	0.038613014	0.1078121	0.3664252	0.06238822	0.42476153	CE	2
CE-19:1	0.079904631	0.0574002	0.3807286	0.07781992	0.4041466	CE	2
CE-19:0	0.029250869	0.0386624	0.1034885	0.06928898	0.75930922	CE	3
CE-20:5	0.085876995	0.2907625	0.212267	0.06735132	0.34374217	CE	4
CE-20:4	0.018051434	0.1629021	0.2162991	0.06511848	0.53762884	CE	2
CE-20:3	0.042914265	0.1649601	0.3078299	0.06639047	0.41790524	CE	2
CE-20:2	0.130961191	0.221213	0.4408269	0.08680128	0.12019758	CE	2
CE-20:1	0.024702816	0.0881374	0.2070504	0.07518924	0.60492014	CE	2
CE-20:0	0.383710475	0.035874	0.2591567	0.08597613	0.23528265	CE	2
CE-21:1	0.09017275	0.1641476	0.087271	0.14240459	0.51600403	CE	1
CE-21:0	0.044094924	0.1415666	0.1356007	0.05890231	0.61983553	CE	1

CE-22:6	0.125229483	0.3147047	0.2431635	0.07302634	0.24387601	CE	4
CE-22:5	0.051545031	0.3516503	0.2849012	0.08245217	0.22945136	CE	4
CE-22:4	0.010781592	0.2484385	0.2105223	0.0623411	0.46791655	CE	4
CE-22:3	0.039938865	0.0525355	0.044466	0.01520897	0.84785072	CE	5
CE-22:2	0.062275612	0.001349	0.0622219	0.00934182	0.86481171	CE	5
CE-22:1	0.16381684	0.0571204	0.1538375	0.11762516	0.50760013	CE	3
CE-22:0	0.130445272	0.0018157	0.0114724	0.03338076	0.82288588	CE	5
TAG44:1(14:0)	0.075321879	0.0196276	0.0462997	0.02292338	0.8358275	TAG	5
TAG44:1(16:0)	0.165370725	0.0330731	0.0022327	0.02765943	0.77166406	TAG	5
TAG44:1(16:1)	0.210004749	0.1498213	0.0002059	0.00368708	0.63628097	TAG	1
TAG46:2(18:2)	0.138833665	0.0130695	0.0453194	0.06355417	0.73922328	TAG	5
TAG46:1(14:1)	0.132294613	0.1057585	0.0007108	0.02391687	0.73731917	TAG	5
TAG48:4(16:1)	0.327645269	0.0003059	0.002566	0.07435084	0.595132	TAG	5
TAG48:3(16:1)	0.468819955	0.022567	0.0138648	0.07561506	0.41913316	TAG	5
TAG48:3(18:2)	0.209918365	0.003345	0.0198501	0.05966084	0.70722571	TAG	5
TAG48:2(16:0)	0.356613458	0.0244455	0.0019155	0.08160919	0.53541632	TAG	5
TAG48:2(16:1)	0.37640307	0.0563911	0.0262662	0.03371018	0.50722949	TAG	5
TAG48:2(18:1)	0.150167388	2.50E-06	0.0271879	0.03309541	0.78954681	TAG	5
TAG48:2(18:2)	0.325842232	0.0160017	0.0172056	0.09661879	0.54433167	TAG	5
TAG48:1(16:0)	0.337080389	0.0388174	0.0004841	0.04518551	0.57843252	TAG	5
TAG48:1(16:1)	0.290173687	0.0973575	2.61E-05	0.04708588	0.56535683	TAG	5
TAG48:1(18:0)	0.105275974	0.025392	0.0135808	0.072358	0.78339322	TAG	5
TAG50:4(16:2)	0.332735917	0.0235165	0.0319335	0.19667315	0.41514091	TAG	3
TAG50:4(18:2)	0.294996151	0.0019092	0.0546883	0.14172074	0.50668562	TAG	3
TAG50:3(16:0)	0.396885425	0.0203925	0.0045434	0.12201535	0.45616333	TAG	5
TAG50:3(16:1)	0.423894533	0.0515583	3.82E-05	0.10260732	0.42190169	TAG	5
TAG50:3(16:2)	0.365710334	0.0313682	0.0199235	0.16395756	0.41904043	TAG	3
TAG50:3(18:1)	0.253559666	4.54E-06	0.02004	0.11610597	0.61028981	TAG	5
TAG50:3(18:2)	0.371248358	0.008035	0.0129102	0.12320728	0.4845991	TAG	5
TAG50:2(16:0)	0.408391282	0.0671194	0.0140266	0.08703132	0.42343137	TAG	5
TAG50:2(16:1)	0.396251377	0.0400033	0.007746	0.0511651	0.50483419	TAG	5
TAG50:2(16:2)	0.234842449	0.0606172	0.0443582	0.13143867	0.52874351	TAG	3
TAG50:2(18:1)	0.32931802	0.0085693	0.0051797	0.04862522	0.60830773	TAG	5
TAG50:2(18:2)	0.376050912	0.072569	0.0498848	0.11613077	0.38536452	TAG	3
TAG50:1(16:0)	0.296794117	0.0498886	0.0111838	0.06159843	0.58053513	TAG	5
TAG50:1(16:1)	0.276414999	0.0590222	0.0001668	0.06693874	0.5974573	TAG	5
TAG50:1(18:0)	0.185338567	0.0361939	0.0109752	0.09181752	0.67567477	TAG	5
TAG50:0(18:0)	0.248808733	0.0505593	0.0134245	0.06727222	0.61993524	TAG	5
TAG51:3(17:1)	0.359599825	0.0194365	0.0187088	0.14687039	0.45538453	TAG	3
TAG51:2(15:0)	0.069149627	0.0003481	0.1472287	0.0700727	0.71320093	TAG	3
TAG51:2(17:0)	0.203893074	0.0352157	0.002253	0.11570546	0.64293278	TAG	5
TAG51:0(17:0)	0.225293854	0.0360512	0.0002044	0.08319156	0.655259	TAG	5
TAG52:6(16:1)	0.209369885	0.1001654	0.1013215	0.1437118	0.44543144	TAG	3
TAG52:6(16:2)	0.098914541	0.0066629	0.1718011	0.14441908	0.5782024	TAG	3

TAG52:6(16:3)	0.076749781	0.0043486	0.0618964	0.22320227	0.63380301	TAG	3
TAG52:6(18:3)	0.112243806	0.087562	0.0853047	0.05580548	0.65908401	TAG	5
TAG52:5(16:0)	0.156433155	0.0552232	0.1009188	0.08882184	0.59860303	TAG	3
TAG52:5(16:1)	0.217260444	0.0010108	0.154375	0.14946887	0.47788494	TAG	3
TAG52:5(16:2)	0.089082523	0.0113898	0.1625784	0.12407942	0.61286979	TAG	3
TAG52:5(16:3)	0.054031654	0.0050734	0.0676674	0.13570749	0.73752	TAG	3
TAG52:5(18:2)	0.19460502	0.0120202	0.1399192	0.13958309	0.51387251	TAG	3
TAG52:5(18:3)	0.171504251	0.0739963	0.0959088	0.10188276	0.55670785	TAG	3
TAG52:4(16:0)	0.265627068	0.0033097	0.1497528	0.10695677	0.47435362	TAG	3
TAG52:4(16:1)	0.136441509	0.0098093	0.1406817	0.13077571	0.58229171	TAG	3
TAG52:4(16:2)	0.098550313	0.0076406	0.1749047	0.16326514	0.55563932	TAG	3
TAG52:4(18:1)	0.16663798	0.0014589	0.0832192	0.11650439	0.63217961	TAG	3
TAG52:4(18:2)	0.262686054	0.0077154	0.1728757	0.11319776	0.44352515	TAG	3
TAG52:4(18:3)	0.177807399	0.0341793	0.0641963	0.10316965	0.62064735	TAG	3
TAG52:3(16:0)	0.33538901	0.0065568	0.1418892	0.08962003	0.426545	TAG	3
TAG52:3(16:1)	0.126017364	0.0100188	0.1565926	0.0656674	0.64170387	TAG	3
TAG52:3(16:2)	0.160803661	0.0281041	0.1197296	0.19557359	0.49578905	TAG	3
TAG52:3(18:1)	0.308461676	0.0074655	0.1591191	0.08904357	0.43591017	TAG	3
TAG52:3(18:2)	0.318454723	0.0100383	0.1518635	0.09980343	0.41984001	TAG	3
TAG52:1(16:1)	0.105732465	0.0453208	0.0530937	0.14431942	0.65153369	TAG	3
TAG53:5(17:1)	0.064603234	0.0016914	0.2100769	0.13370789	0.58992049	TAG	3
TAG53:5(18:3)	0.006891781	0.0595996	0.1120496	0.08767187	0.73378719	TAG	3
TAG53:3(16:0)	0.247149416	0.0502591	0.1030497	0.10121204	0.49832971	TAG	3
TAG53:3(19:1)	0.219993916	0.0389474	0.1219096	0.10119496	0.51795414	TAG	3
TAG53:2(17:1)	0.112206234	0.0312435	0.1435845	0.07536335	0.63760238	TAG	3
TAG53:2(19:0)	0.184889254	0.0627741	0.1083046	0.09345283	0.55057925	TAG	3
TAG53:2(19:1)	0.220182098	0.026096	0.0325223	0.04816521	0.67303441	TAG	5
TAG54:8(22:6)	0.330862255	0.0249039	0.1144916	0.0629629	0.46677936	TAG	3
TAG54:7(20:4)	0.248674833	0.00631	0.105222	0.14910841	0.49068474	TAG	3
TAG54:7(20:5)	0.176046724	0.0111869	0.0834373	0.08243862	0.64689056	TAG	3
TAG54:7(22:6)	0.350930216	0.0637714	0.0619321	0.02759714	0.4957691	TAG	5
TAG54:6(18:2)	0.099153177	0.0096233	0.2677591	0.11479665	0.50866778	TAG	2
TAG54:6(18:3)	0.07653501	0.0382597	0.1821751	0.10167338	0.60135684	TAG	3
TAG54:6(20:4)	0.324536052	0.0477706	0.0671323	0.15458554	0.40597552	TAG	3
TAG54:5(16:0)	0.347447762	0.0600451	0.0676464	0.12340796	0.40145279	TAG	3
TAG54:5(16:1)	0.217429811	0.0962515	0.128649	0.15659759	0.40107209	TAG	3
TAG54:5(16:2)	0.141064876	0.0566046	0.2911481	0.14771747	0.36346494	TAG	2
TAG54:5(18:1)	0.113642763	0.0153533	0.2254436	0.12328904	0.52227128	TAG	2
TAG54:5(18:2)	0.104068341	0.0336481	0.2439635	0.11645485	0.50186517	TAG	2
TAG54:5(18:3)	0.110922697	0.0116064	0.1214555	0.09470886	0.66130656	TAG	3
TAG54:5(20:4)	0.319135933	0.062333	0.030301	0.11287267	0.47535737	TAG	5
TAG54:4(16:0)	0.296493614	0.099524	0.1772216	0.12761089	0.29914983	TAG	3
TAG54:4(16:1)	0.134297633	0.094429	0.2368203	0.09587722	0.43857589	TAG	2
TAG54:4(16:2)	0.143209129	0.0585918	0.2910515	0.14318459	0.36396304	TAG	2

TAG54:4(18:0)	0.199279555	0.0290617	0.2554041	0.12922556	0.38702916	TAG	2
TAG54:4(18:1)	0.13988449	0.0171637	0.2414079	0.08998464	0.51155932	TAG	2
TAG54:4(18:2)	0.142295967	0.0207513	0.2407886	0.08687298	0.50929121	TAG	2
TAG54:4(18:3)	0.15814698	0.0007734	0.1361755	0.11224626	0.59265789	TAG	3
TAG54:3(16:0)	0.242096485	0.0786585	0.194545	0.08035703	0.40434294	TAG	3
TAG54:3(18:1)	0.155692854	0.0076252	0.1529487	0.04346853	0.64026481	TAG	3
TAG54:3(18:2)	0.209703626	0.0244536	0.2354685	0.09462908	0.43574516	TAG	2
TAG54:2(18:0)	0.172479636	0.0083353	0.1589194	0.0454301	0.61483557	TAG	3
TAG54:2(18:2)	0.225050868	0.0412107	0.2351558	0.12028661	0.37829602	TAG	2
TAG55:6(19:3)	0.240284647	0.0103916	0.2387007	0.10312285	0.40750027	TAG	2
TAG55:5(18:2)	0.095635836	0.0298085	0.3045795	0.1659583	0.40401786	TAG	2
TAG56:8(20:6)	0.165401157	0.0147088	0.1980954	0.04340239	0.57839222	TAG	3
TAG56:8(18:2)	0.280324104	0.0271556	0.2438757	0.16506341	0.28358121	TAG	2
TAG56:7(18:3)	0.116779809	0.0011788	0.1328524	0.13582216	0.61336686	TAG	3
TAG56:7(22:5)	0.225356329	0.0126698	0.1182627	0.12825778	0.51545342	TAG	3
TAG56:6(18:2)	0.177874158	0.0420186	0.245102	0.17940979	0.35559547	TAG	2
TAG56:6(22:4)	0.301500693	0.0707728	0.1196056	0.15705523	0.35106564	TAG	3
TAG56:6(22:5)	0.1814553	0.0221283	0.0909925	0.09293985	0.61248408	TAG	3
TAG56:5(18:1)	0.171262919	0.0634357	0.2407076	0.14582891	0.37876487	TAG	2
TAG56:5(18:2)	0.131120871	0.0723429	0.2459912	0.11434997	0.436195	TAG	2
TAG56:5(20:2)	0.101332233	0.0962659	0.3211292	0.14107159	0.34020106	TAG	2
TAG56:5(22:4)	0.216078485	0.0738867	0.0459082	0.12951095	0.53461569	TAG	3
TAG56:4(20:0)	0.100129392	0.0898046	0.1399678	0.08982432	0.58027388	TAG	3
TAG56:4(20:2)	0.125483132	0.0802634	0.2485359	0.11165172	0.43406588	TAG	2
TAG58:8(22:5)	0.089598419	0.0396975	0.1849098	0.0970925	0.58870184	TAG	3
TAG58:7(20:4)	0.161907992	0.0929349	0.185636	0.11287278	0.4466483	TAG	3
TAG58:7(22:5)	0.067275551	0.0387665	0.1223198	0.07467806	0.69696011	TAG	3

The table provides the explained variance (R2) of each serum lipidomic traits by null_model (including BMI, FPG, and SBP), sex, age, and age-by-sex interaction estimated using GAMs. In addition, it shows the position of each lipoprotein in the hierarchical clustering and the cluster group, as presented in Supplementary Figure S3.
